# Preparation of Biomass Waste-Derived Carbon Dots by the Thermal Degradation Process

**DOI:** 10.1021/acsomega.4c10119

**Published:** 2025-05-27

**Authors:** Wiktoria Matyjasik, Krzysztof Matus, Olga Długosz, Jolanta Pulit-Prociak, Marcin Banach

**Affiliations:** † CUT Doctoral School, Faculty of Chemical Engineering and Technology, Department of Chemical Technology and Environmental Analytics, 49571 Cracow University of Technology, Warszawska St. 24, 31-155 Cracow, Poland; ‡ Institute of Engineering Materials and Biomaterials, 49569 Silesian University of Technology, Konarskiego 18A, 44-100 Gliwice, Poland; § Faculty of Chemical Engineering and Technology, Department of Chemical Technology and Environmental Analytics, 49571 Cracow University of Technology, Warszawska St. 24, 31-155 Cracow, Poland

## Abstract

Agricultural and food industry waste biomass is a material with diverse origins and a resulting variety of physicochemical characteristics and compositions. Because of its source, it is characterized by a high carbon content, and so it has been considered a worthwhile substrate for the synthesis of carbon dots (CDs). Therefore, waste biomass with high availability in Central Europe was selected as substrates for CDs synthesis: apple pomace, rapeseed pomace, and potato peelings. These materials are characterized by varying the composition and nitrogen content. The synthesis of CDs by thermolysis was investigated. The obtained CDs were examined by UV–vis spectrophotometry, spectrofluorimetry, Fourier transform infrared spectroscopy, high-resolution transmission electron microscopy, and X-ray diffraction. The CDs synthesis yield (SY) and fluorescence quantum yield (QY) were determined. This paper proves that CDs can be synthesized efficiently by thermal degradation in a solvent-free process using plant waste biomass. Despite the varying compositions of the substrates, CDs with similar emission characteristics were achieved. CDs obtained from apple pomace and potato peelings have a crystalline structure and size of 4–5 nm, while CDs obtained from rapeseed pomace have an amorphous structure and size of 11 nm. CDs derived at 260 °C from potato peelings are characterized by the strongest fluorescence among synthesized materials, a QY of 14.5%, with 12.3% SY. The potential of the obtained CDs as an optical biosensor was investigated against a variety of metal ions and water pollutants. The CDs obtained from potato peelings can be effectively used in the detection of Fe^3+^ ions (linearity range 12.5–1250 μM) and ibuprofen (linearity range 0.25–5 mM).

## Graphical abstract

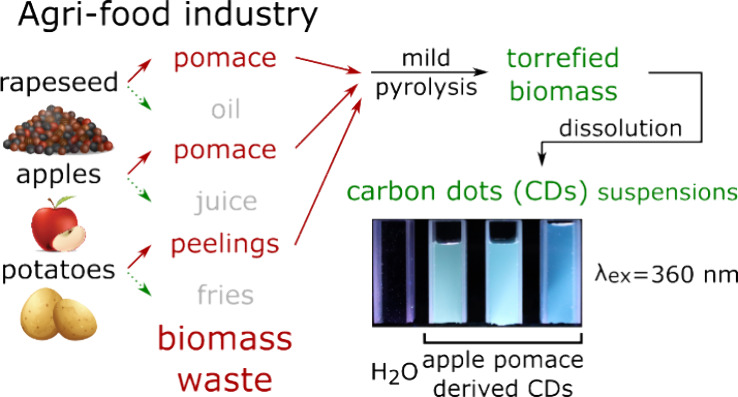

## 1 Introduction

Carbon dots (CDs) are a relatively young type of carbon nanomaterial, discovered by Xu et al. in 2004.^1^ They are established as quasi-spherical carbon nanostructures with an average size of <10 nm, usually zero-dimensional, composed of a carbon skeleton and functional groups on their surface, and with fluorescent properties.^2–5^ CDs could be synthesized by different methods, also in solvent-free processes, of which one is thermal degradation. In this method, both pure chemical compounds (e.g., organic acids,^6,7^ saccharides,^8,9^ amino acids^10,11^ ), as more complex (e.g., starch,^12^ microcrystalline cellulose^13^), whether substances of natural origin (fruits,^14^ seeds,^15^ shells,^16^ husks,^17^ peels,^18^ etc.). Substrates in thermal processes undergo transformations, such as polymerization, decomposition, condensation, graphitization (carbonization), and surface passivation.^3,4^ In this study, the synthesis is conducted by low-temperature pyrolysis (also known as mild pyrolysis or torrefaction). It is a thermal treatment of biomass performed at temperatures between 200 and 300 °C with limited oxygen and low heating rates. The process is used to remove and reduce the oxygen content of the biomass. It is a pretreatment process of waste biomass used to improve the quality of bio-oil or biochar obtained in the subsequent pyrolysis process.^19–22^ Thermal degradation in both air and inert gas atmospheres makes it possible to achieve CDs efficiently and on a large scale.^27^ Torrefaction was deemed to be under-researched in carbon dot synthesis; the research is mainly focused on biomass pyrolysis for sustainable biochar synthesis.

In 2021, the FAO determined that the world’s total agricultural land area is 4.8 billion hectares, about one-third of the Earth’s land area, of which cropland occupies 1.6 billion hectares.^23^ In 2020, in the European Union, 58% of farms specialized in crops, using an area of 81 million hectares (52% of the farm area). Among field crops, farms producing, in particular, root crops (including potatoes) accounted for 18% of all farms, and those producing, in particular, oilseeds (including rapeseed) accounted for 16% of farms.^24^ FAO put total world apple production in 2022 at 95.8 million tons, with China producing 47.6 million tons of apples,^25^ then 12.6 million tons of apples were produced in the EU (34% of which were produced in Poland).^25,26^ In the case of rapeseed and turnip rape, the FAO put total world production in 2022 at 87.2 million tons, with the largest volume of production in the EU, where 19.4 million tons were produced (including 18% in Poland).^25,26^ Total world potato production in 2022 was set at 374.8 million tons, of which 95.6 million tons were produced in China and 56.2 million tons in India.^25^ Potatoes are one of the two most widely grown root vegetables in the EU, with a harvest of 47.5 million tons (including 13% in Poland).^25,26^

Waste from the agro-food industry is difficult to manage because of the significant volume of production, not only regionally but also globally, the variability of composition, seasonality of production, and the variable supply associated with the varying size of farms/production companies. The agricultural and food industry waste biomass is a group of materials with a wide variety of physicochemical characteristics and composition, which are associated with a highly diverse source of origin. Vegetable waste is characterized by a composition rich in cellulose, lignocellulose, and lignin. In addition, depending on the source of the waste, the season, and other external factors, the content of proteins, fats, sugars, and other substances can vary, which can affect the difficulty of its management. Dried apple pomace contains mainly fructose (11.5–49.8 g/100 g dry mass (DM)), glucose (2.5–22.7 g/100 g DM), dietary fiber (26.8–82 g/100 g DM), proteins (1.2–6.91 g/100 g DM) as well as fats (0.26–8.49 g/100 g DM).^27^ Nawirska and Uklańska examined pomace from apples of the Szampion variety for the composition of dietary fiber, which consists of cellulose (16.1 g/100 g DM), hemicelluloses (9.37 g/100 g DM), and lignins (5.8 g/100 g DM).^100^ Rapeseed pomace mainly contains proteins (25.8–35.9 g/100 g DM), fats (11.3–22.9 g/100 g DM), and dietary fiber (7.1–8.8 g/100 g DM).^28^ Rommi with his team studied the rapeseed pomace carbohydrate composition (35.4 g/100 g DM), which consists of, among other things, glucose (14.7 g/100 g DM), fructose (7.4 g/100 g DM), insoluble carbohydrates (19.8 g/100 g DM), and lignin (18.8 g/100 g DM).^29^ Potato peelings contain mainly starch (16.8–67 g/100 g DM), nonstarch polysaccharides (22.5–46.9 g/100 g DM), lignin with suberin (21–47.7 g/100 g DM), and proteins (2.1–18.5 g/100 g DM).^30–33^

Plant waste biomass has been identified as a worthwhile substrate for carbon dot synthesis. The management of waste biomass from plants with significant production volumes in Central Europe is under-researched; most of the scientific research published to date is focused on plant waste, the management of which is a problem in other regions of the world and whose production volumes in Central Europe are low, and are therefore limited feedstocks. In addition, the use of thermolysis to synthesize CDs from complex materials is limited; most of the published work involves processing biomass by hydrothermal carbonization in solvent processes, mainly from expensive pure chemical reagents. The primary objective of this study was to verify the feasibility of synthesizing carbon dots using plant waste biomass from the agrifood industries of various characteristics by thermal decomposition in a solvent-free process. This manuscript presents an attempt to develop a new approach to CDs synthesis planning, considering the results of thermal analysis in determining process operating temperatures, using materials with diverse compositions resulting in different thermal decomposition characteristics. The use of thermal analysis in the planning of syntheses makes it possible to identify in a natural lignocellulosic material the steps involved in the decomposition of cellulose, hemicellulose, and lignin, taking into account the complex chemical composition and thermal effects of the processes occurring, making it possible to avoid overdecomposition of the sample and the resulting yield of mainly biochar.

## 2 Materials and Methods

### 2.1 Chemicals and Materials

Rapeseed pomace (Brassica napus) in the form of pellets was received from a local producer of rapeseed oil obtained using a pressure press. Potatoes (Solanum tuberosum L.) and apples (Malus domestica var. Szampion) were purchased from the local grocery store. Potatoes were Potatoes were peeled, and the skins were collected and dried in a drying oven at 80 °C, which were collected and dried in a drying oven at 80 °C. Seed nests were cut from the apples, then juice was squeezed from the seedless apples using a slow-speed juicer to reach waste apple pulp, which, along with the seed nests, was collected and dried in a dryer at 80 °C. After drying, the waste biomass for the study was prepared by grinding the material using a knife grinder and then running it through a laboratory sieve with a mesh size of 250 μm (MULTISERW-Morek, Poland) without concentration. Grinding and sieving were repeated until all the prepared material successfully passed through the specified sieve, resulting in biomass particles of uniform size <250 μm. Apple pomace and potato peelings prepared in this way were visually homogeneous powder, with uniform color throughout. The rapeseed pomace prepared in this way was not a completely homogeneous powder - the powder consists of two types of particles, which were thoroughly mixed before weighing. A homogeneous powder was not possible with the chosen grain size; however, due to the intention not to interfere with the thermal process relative to the other materials, because of reducing the grain size, the material was tested in this form, being mixed carefully before weighing. The materials prepared in this way were stored in airtight containers and used for the study.

Hydrochloric acid HCl (35–38%, POCH, Poland) and sodium hydroxide NaOH micropills (p.a. grade, POCH, Poland) were used to adjust the pH. To determine the fluorescence quantum yield (QY) of the obtained CDs by the relative method, fluorescence standard quinine hemisulfate salt monohydrate (BioReagent, 99–101%, Merck, Germany) was used. For the dissolving of quinine sulfate, sulfuric acid H_2_SO_4_ (96%, Carl Roth, Germany) was used.

Metal salts: calcium chloride (≥93%, Merck, Germany), cobalt­(II) chloride hexahydrate (p.a. grade, CHEMPUR, Poland), chromium­(II) chloride (90%, ACROS Organics, USA), copper­(II) chloride (97%, Merck, Germany), iron­(II) chloride tetrahydrate (p.a. grade, ≥99%, Merck, Germany), iron­(III) chloride (97%, Merck, Germany), mercury­(II) chloride (≥99,5%, Merck, Germany), potassium chloride (BioXtra, ≥99%), lithium chloride (≥99%, POL-AURA, Poland), magnesium chloride (≥98% Merck, Germany), nickel­(II) chloride hexahydrate (BioReagent, Merck, Germany), lead­(II) chloride (98%, Merck, Germany), and zinc chloride (p.a. grade, ≥98%, Merck, Germany) were used to verify the sensitivity of the CDs to the metal content of the solution. Bioactive substances: ibuprofen (commercial grade), paracetamol (commercial grade), chloridazon (≥98%, Merck, Germany), atrazine (≥98%, Merck, Germany), and diuron (≥98%, Merck, Germany), were used to verify the interaction between the tested substance and the CDs. All chemicals were used without further purification. The deionized water (type II pure water, 0.05 μS/cm) used in the experiments was provided by an HLP10 (Hydrolab, Poland) demineralizing system.

To preliminarily purify product suspensions from solid particles, Cellulose qualitative filters (Munktell filter paper, wet-strengthened grade 1288 with 12–15 μm retention and 1289 with 8–12 μm retention, Ahlstrom-Munksjö, Finland) were used; in the next step, a hydrophilic PVDF membrane with 0.1 μm pore size (Merck, Germany, Durapore Membrane Filter) was used. Standard regenerated cellulose dialysis tubes with 3.5 kDa molecular weight cutoff (MWCO) (Spectrum Laboratories, USA, Spectra/Por 3) were used for the final purification of CDs suspensions from salt residues and small water-soluble particles.

### 2.2 Substrate Characteristics

To determine the elemental composition of the plant wastes used, CHNS elemental analysis was performed by using a CHN/CHNS EuroEA3000 analyzer (EuroVector, Italy) with an M2P analytical microbalance (Sartorius, Germany). The determination was carried out using the Dumas dynamic combustion method, followed by chromatographic separation of the resulting gas fractions (N_2_, CO_2_, H_2_O, SO_2_), followed by their analysis using a katharometer. Oxygen content was determined as a difference relative to the other elements.

Thermal analyses (DTA/TG) were performed to determine the thermal characteristics of the substrates used in the synthesis. The measurements were performed with a Jupiter STA 449F3 instrument (Netzsch, Germany) at a temperature range of 30–1000 °C, in a synthetic air atmosphere, with an increase in temperature of 10 K/min, in an Al_2_O_3_ crucible. The analysis made it possible to identify the course of transformations occurring in the material under the influence of temperature and to determine the temperature range of the syntheses conducted on their basis. As a result of the analysis of TG, DTA and DTG diagrams for biomass waste and for fructose, starch and cellulose, the maximum temperatures of the conducted process were predetermined, to avoid, as far as possible, large (>50%) and rapid (>10%/min.) mass losses, to avoid uncontrolled and excessive decomposition of the material leading to too intense carbonization, defined as an undesirable process. The selected temperatures were verified by conducting trial processes, temperatures were chosen based on weight loss and color changes (visible browning, but before blackening). If the product met the assumptions associated with the specified maximum mass loss, the temperature was determined as the maximum process temperature. Syntheses were also performed at temperatures 20 and 40 °C lower than the designated maximum temperature, respectively. Temperatures in 20 °C increments against each other were chosen to reduce the number of samples to be made due to the time-consuming nature of the process, while allowing the widest possible temperature range to be covered and to identify the most preferred parameters. The processes were conducted according to the methodology given in Section 2.3.

### 2.3 Synthesis of Waste Biomass-Derived Carbon Dots

Figure 1 shows a simplified scheme of the synthesis process from biomass extraction to the final CD solution, including the preparation of the synthesis substrates described in Section 2.1.

**Figure 1:**
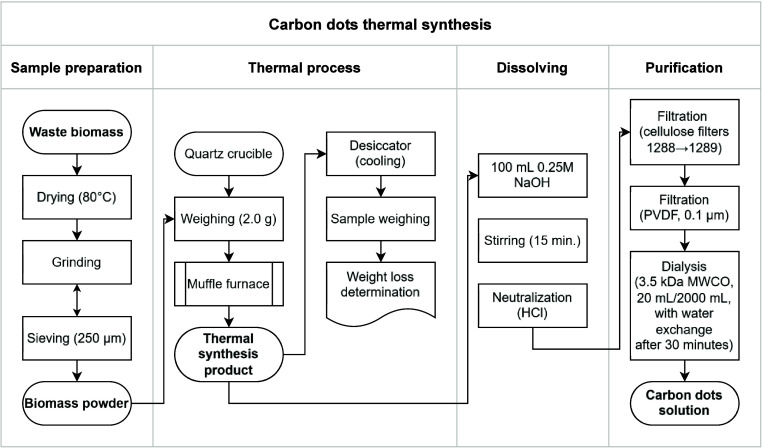
Full scheme of CDs synthesis from substrate acquisition and preparation, through the process of thermal synthesis, dissolution, and purification of the obtained CDs.

To synthesize carbon dots by thermal degradation, 2.0 g of previously prepared material (apple pomace, rapeseed pomace, and potato peelings) was weighed into a 30 mL quartz crucible with a lid. The crucible with the sample was transferred to an SM-2002 muffle furnace (Czylok, Poland).

The processes were conducted according to the synthesis plan shown in Table 1, at the assumed target temperature *T*, at the assumed target temperature *T*, in a semiclosed system, under a self-generated atmosphere, achieving an average mass loss of *d*_*m*avg_. The oven was heated to *T*-30 °C at a rate of 3 °C/min, after which the temperature was held constant at *T*-30 °C for 5 min. Then, to the final temperature of *T*, the oven was heated at a rate of 1.5 °C/min. After reaching the set temperature, the material stayed in the oven for 10 min (*t*). Once the specified process time had elapsed, the crucible containing the sample was moved to a desiccator for cooling. After cooling, the sample was weighed to determine weight loss (*d*_*m*_) in the conducted process. By repeating the processes three times, the weight loss of the material was used to verify the repeatability and reproducibility of the process. After the obtained materials were crushed in an agate mortar, the material from a single thermolysis process was dissolved in 100 mL of 0.25 M sodium hydroxide solution. The suspension was stirred magnetically for 15 min, after which it was neutralized with HCl solutions. To remove solid residues, the resulting suspension was filtered under reduced pressure using cellulose filters 1288 and 1289 grade successively and then using a 0.1 μm pore membrane made of PVDF. The resulting filtrate was dialyzed against deionized water using 3.5 kDa MWCO membranes in a two-step process to remove small byproducts and ions involved in leaching CDs from the pyrolyzed material. Twenty mL of the achieved solution was dialyzed for 30 min in 2 L of deionized water with continuous stirring at 300 rpm, after which the external solution was exchanged for a fresh portion of water and dialysis was carried out for another 30 min. The resulting CD suspension is the final product.

**Table 1:** Summary of CD Synthesis Parameters, Labels of the Obtained Products, and Average Weight Loss of the Material

no.	material	*T* [°C]	CDs labeling	*d*_*m*avg_ [%]
1	apple pomace	180	**J-180–10**	24.8% ± 0.51%
2	apple pomace	200	**J-200–10**	29.4% ± 0.54%
3	apple pomace	220	**J-220–10**	32.6% ± 0.83%
4	rapeseed pomace	220	**R-220–10**	13.3% ± 0.45%
5	rapeseed pomace	240	**R-240–10**	20.3% ± 0.73%
6	rapeseed pomace	260	R-260–10	25.7% ± 0.73%
7	potato peelings	220	**Z-220–10**	8.5% ± 0.86%
8	potato peelings	240	**Z-240–10**	25.1% ± 1.47%
9	potato peelings	260	**Z-260–10**	40.9% ± 0.89%

In order to better understand the nature of the studied process, for potato peelings, the parameters of the conducted processes were extended, relative to the basic processes for potato, listed in Table 1. The temperature range was increased from 220–260 to 220–340 °C, and a 30 min residence time of the sample at the assumed temperature in the oven was added. Table S1 presents the parameters of the processes conducted, along with the corresponding weight losses. The processes were performed according to the instructions provided above.

### 2.4 Determination of Process Yields

In order to determine the yield of CDs (SY_CDs_), the density of the obtained solutions after dialysis (*d*), the dry weight (*d_m_*), and based on these, the concentrations (*C*_CDs_) of the obtained CDs were determined. The volume of all solutions after neutralization was estimated to be 112.5 ± 2.5 mL. It was assumed that only CDs are present in solution after dialysis, neglecting the presence of other water-soluble compounds that were not removed in the process.

Density (*d*) was determined by pycnometer, using 10 mL of Gay-Lussac pycnometers, at ambient temperature, in triplicate, after which the average density was determined along with standard deviations. Dry weight (*d_m_*) was also determined in triplicate, using 30 mL porcelain crucibles previously roasted to constant weight. About 2 mL of solution was poured into the preweighed crucible, the crucibles were placed in a 105 °C dryer for 24 h, after which the crucibles were placed in a desiccator to cool, weighed again, and the average dry mass content in mg/g of solution was determined, along with standard deviations. The next step was to determine the concentration of CDs (*C*_CDs_) in the tested solutions, according to the formula $C_{\mathrm{CDs}}=d\cdot m\left[\frac{\mathrm{mg}}{\mathrm{mL}}\right]$ . To determine the yield of the CD synthesis process against the weight of product obtained after the thermal process (*m_t_*), the theoretical concentration (*C_t_*) was calculated according to the formula $C_{t}=\frac{m_{t}}{V_{t}}\left[\frac{\mathrm{mg}}{\mathrm{mL}}\right]$ . The yield of CDs was then determined according to the formula ${\mathrm{SY}}_{\mathrm{CDs}}=\frac{C_{\mathrm{CDs}}}{C_{t}}[\%]$ .

### 2.5 Physicochemical Characteristics of the Obtained Materials

To determine the absorption spectra of the obtained samples and the absorbance of the prepared dilutions, UV–vis spectroscopy was used. The measurements were performed in a quartz cuvette of 10 mm optical path length, using the Rayleigh UV-1800 (Beijing Beifen-Ruili Analytical Instrument Co., Ltd., China) spectrophotometer in the 200–800 nm range. For further fluorescence analyses, derived CDs solutions were diluted before measurements to achieve an absorbance under 0.1, which was diluted twice at a 350 nm wavelength.

Examination of the fluorescence of the obtained CDs was conducted using a microplate reader, SpectraMax M2 (Molecular Devices, USA). For fluorescence measurements, 3 solutions were used for each of the samples–concentrated (obtained after dialysis), with absorbance under 0.15 and with absorbance under 0.15 diluted twice were used. The fluorescence was examined at three points to determine the relationship between absorbance and fluorescence and whether concentration quenching occurs. Based on the data obtained, the fluorescence area at excitation with 350 nm light was plotted against absorbance at 350 nm for each sample, and linear trend curves were determined. For the synthesized products, it was assumed that the most desirable excitation wavelength should be in the near-ultraviolet range (300–400 nm) for utility reasons. Fluorescence spectra analyses were performed with λ_ex_ = 350 nm excitation wavelength, which allows for maximum emission peaks within the assumed emission range (visible, >400 nm).

The quantum yield (QY) of fluorescence was measured by the relative method, following the methodology described by Würth et al.,^34^ with modification resulting from fluorescence measurements for each sample at three concentrations. Analyzes were performed with a quinine sulfate (QS) at a concentration below 1 mg/L (absorbance under 0.15), dissolved in 0.5 mol/L H_2_SO_4_, excited by light of λ_ex_ = 350 nm. With the data obtained for each QS solution, the fluorescence area versus absorbance curves were plotted, and a linear trend curve was determined (Figure S1). The CD quantum yield was determined using fluorescence spectra gained under excitation with light of λ_ex_ = 350 nm, and the area was determined in the range of 400–600 nm.

The QY was determined according to the formula below:

$${\mathrm{QY}}_{x}={\mathrm{QY}}_{\mathrm{ref}}\cdot \frac{a_{x}}{a_{\mathrm{ref}}}\cdot \frac{\eta_{x}^{2}}{\eta_{\mathrm{ref}}^{2}}$$ 1

where QY–quantum yield (0.6 for QS with λ_ex_ = 350 nm^55^); *a*–coefficient a of the linear trend curve; η–refractive index of the solvent (in the UV region −1.348 for water,^35^ 1.347 for 0.5 mol/L H_2_SO_4_ ^36^). The subscript *x* denotes the sample evaluated; the subscript ref denotes the fluorescence standard used.

Fluorescence photostability analysis was carried out for selected samples: samples were exposed to a UV LED lamp at 360–370 nm (Liteon LTPL-C034UVD365, Taiwan) for 1 h, after which the fluorescence spectrum was measured at an excitation wavelength of 350 nm and the % decrease in fluorescence was determined.

The shape and morphology were studied by HR-TEM (high-resolution transmission electron microscopy), using FEI Titan 80–300 system (FEI, USA). Selected CDs for analysis were obtained through the processes of J-220, R-240, and Z-260. ImageJ software was used for calculating the diameter and *d*-spacing of CDs of TEM. ImageJ (1.48 (v) software was used to estimate the particle size distribution of the C-dots from the TEM images.

The XRD method was used to determine the crystallographic structure of the obtained CDs. Selected CDs for analysis were obtained through the processes of J-220, R-240, and Z-260. Samples were dried at 105 °C for analysis. The device used was a SmartLab SE (Rigaku) instrument, using a Cu Kα radiation source, in the range of 2θ angle, from 5° to 80°. The *d*-spacing of nanoparticle crystallites was determined from the Bragg equation:

$$d_{\mathrm{spacing}}=\frac{n\lambda }{2\sin\;\Theta }$$

where *d*_spacing_–interplanar spacing, *n* = 1–order of diffraction, λ = 0.154 nm–Roentgen radiation wavelength, and θ–angle formed by the ray falling on an atomic plane.

The Fourier transform infrared spectroscopy (FT-IR) was used for the comparison between substrates and products obtained after thermal processes and between a group of obtained CDs, by identification of defined bands of molecular vibrations, which leads to conclusions about the presence of specific functional groups in the tested compound. CD samples were dried at 105 °C for analysis. All materials were dried at 80 °C overnight before analysis. FT-IR measurement was carried out on a Nicolet iS5 spectrometer equipped with a diamond ATR attachment (Thermo Fisher Scientific, USA). Spectra were recorded in the range of 4000–400 cm^–1^, with a resolution of 0.48 cm^–1^.

### 2.6 Optical Detection of Various Substances

To determine the potential for detecting biologically active substances and metal ions, carbon dots (CDs) synthesized via the Z-260 process were utilized. The solutions were prepared as described in Section 2.3. Fluorescence measurements were conducted at 370 nm due to the narrower fluorescence peak observed at this wavelength compared to that at 350 nm, where a broader peak with a larger area is obtained. Fluorescence was measured in at least duplicate samples, and changes were analyzed.

First, the effect of pH on fluorescence was studied. The pH range of 2–12 was investigated, with steps of around 1 unit, with adjustments made using 0.05 and 0.1 M solutions of HCl and NaOH (initial pH approximately 6.7). To 1.5 mL of the sample, an appropriate amount of acid/base (<0.1 mL) was added, thus neglecting any fluorescence changes due to dilution. The pH was measured using a pH meter, and after stabilizing the pH at the desired level, the fluorescence of the sample was measured at 370 nm. The experiments were performed twice.

The substances tested included metal ions from chloride salts (calcium, cobalt­(II), chromium­(II), copper­(II), iron­(II), iron­(III), mercury­(II), potassium, lithium, magnesium, nickel­(II), lead­(II), and zinc) at a base concentration of 6.25 mM, as well as compounds such as paracetamol, ibuprofen, chloridazon, atrazine, and diuron at a base concentration of 20 mg/L, selected due to their relevance as water pollutants. For the ions, 3 consecutive concentrations were tested (base, 5x dilution, and 10x dilution); for the substances tested, 2 concentrations were tested (base and 10x dilution).

For the detection process, a 1:1 (v/v) mixture of the CD solution and the tested substance solution was prepared. Changes in fluorescence intensity (enhancement or quenching) relative to the pure CDs solution were analyzed, and substances displaying interactions with the CDs were further investigated. Changes in the shape and area of the peaks obtained were observed and analyzed. Due to the strongest fluorescence quenching in a specific group of compounds, Fe^3+^ ions and ibuprofen were tested, respectively.

A more detailed fluorescence study was conducted for Fe^3+^ ions and ibuprofen (IBU) using solutions with the concentrations specified in Table 2. Fluorescence was measured in at least triplicate. The fluorescence peak area of the mixture of CDs with the test substance was analyzed against the fluorescence peak area of CDs mixed in a ratio of 1:1 (v/v) with deionized water. Concentration ranges useful for optical detection using fluorescence were determined, and curves were drawn for the dependence of the fluorescence intensity on the concentration of the test substance. LOD value was determined using the formula 3*sd*_0_/*k*, where *sd*_0_ is the standard deviation of the blank signal (water), the *sd*_0_ value is equal to 0.048%, and *k* is the slope of the calibration curve.

**Table 2:** Investigated Concentrations of Substances Affecting CD Fluorescence

Fe^3+^		IBU
*C_m_* [mM]	*C* [mg/L]	*C* [mg/L]
6.250	349.03	20
3.125	174.52	15
2.500	139.61	10
1.250	69.81	8
1.000	55.85	5
0.625	34.90	2
0.3125	17.45	1
0.1250	6.98	0.25
0.0625	3.49	
0.0125	0.70	
0.00125	0.07	

## 3 Results and Discussion

### 3.1 Elemental Analysis of Substrates

The results of the elemental analysis are summarized in Table 3. The content of carbon, crucial to the synthesis of CDs in the materials studied, is in the range 43–50%, with the highest value for rapeseed pomace. As cited in the introduction, apple pomace and potato peelings possess distinct chemical compositions, despite their similar overall elemental composition. The nitrogen content of the raw materials used in the study is characterized by significant variation, with rapeseed pomace being the richest. This is because of the high protein content relative to that of the other wastes. A lower oxygen content can be observed in rapeseed pomace than in the other materials, which may be the result of the fatty acids remaining in the material after pressing, characterized by long hydrocarbon chains. None of the materials showed the presence of sulfur (below the limit of quantification).

**Table 3:** Summary of the Results of the Elemental Analysis of the Substrates Used in the Study

material	C [%]	H [%]	N [%]	O [%]	S [%]
apple pomace	43.2 ± 0.56	9.3 ± 0.04	0.4 ± 0.01	47.2 ± 0.61	0.0
rapeseed pomace	50.2 ± 0.10	10.6 ± 0.11	4.9 ± 0.11	34.3 ± 0.31	0.0
potato peelings	42.6 ± 0.49	8.6 ± 0.06	2.0 ± 0.02	46.8 ± 0.45	0.0

### 3.2 Thermal Analysis of Substrates

In Figure 2A, the results of thermal analysis of the apple pomace sample are presented, a peak of endothermic transformation, which was observed with a maximum at 100 °C, associated with evaporation of residual moisture. In the range of about 150–260 °C, another stage of transformation can be observed, with a total mass loss of approximately 35% and a mass loss maximum of about 8%/min, at 208 °C. A mild peak of endothermic transformation appears on the DTA curve, identified with the breakdown of sugars present in the material. Zlatanović and his team studied the thermal decomposition of apple pomace flours in an inert gas atmosphere when heated at 5 °C/min. The researchers observed the occurrence of a similar peak in weight loss in the materials under investigation, which they identified as the decomposition of monosaccharides and simple polysaccharides.^37^ In a further stage of the process, in the range of about 260–335 °C, decomposition results in a further 10% weight loss, two overlapping transformations occur, and the weight loss is slowed relative to the previous stage, with the first transformation visible as an inflection seen in the peak of the second transformation, whose maximum is about 4% of the sample weight per minute at about 324 °C. Zlatanović et al. identified an analogous composite peak of two overlapping thermal effects as related to the thermal decomposition of hemicellulose and cellulose, as well as pectins and proteins.^37^ Guerrero and his team performed a thermal analysis of apple pomace, under an inert gas atmosphere with varying heating rates, also determined that an analogous complex peak is associated with the thermal decomposition of hemicellulose and cellulose, as a result of depolymerization, and dehydration, among others, leading to the formation of gaseous products, as well as the polymerization, carbonylation, carboxylation and transglycosylation that occurs.^38,39^ The next stage of the process occurs in the range of about 335–410 °C and is associated with a slowing exothermic transformation, leading to a mass decrease of approximately 12% throughout the stage. The exothermic decomposition under the conditions studied is associated with the combustion of the test material with air. In the further part of the process, it is possible to distinguish another stage, in which a mass loss of another about 5% occurs, with a further decrease in the rate of mass loss, in the range of about 410–445 °C, associated with an exothermic transformation with a maximum at about 434 °C, this stage smoothly passes into the next one in the range of 445–620 °C consequently leading to a mass of about 1.9% of the initial mass of the sample, this loss is associated with an endothermic transformation with a constant rate of mass loss of about 1.75%/min. The transformation is associated with the decomposition of, in particular, lignin, the thermal decomposition of which occurs in the range of 160–900 °C, resulting in the formation of solid residues.^40^ The significant decrease in weight loss at >400 °C was determined by Guerrero et al. to be due to the characteristic decomposition rate of lignin.^38^ At >620 °C, the sample undergoes a final, slowed exothermic transformation, reaching a constant mass of about 1.5% of the sample’s initial mass.

**Figure 2:**
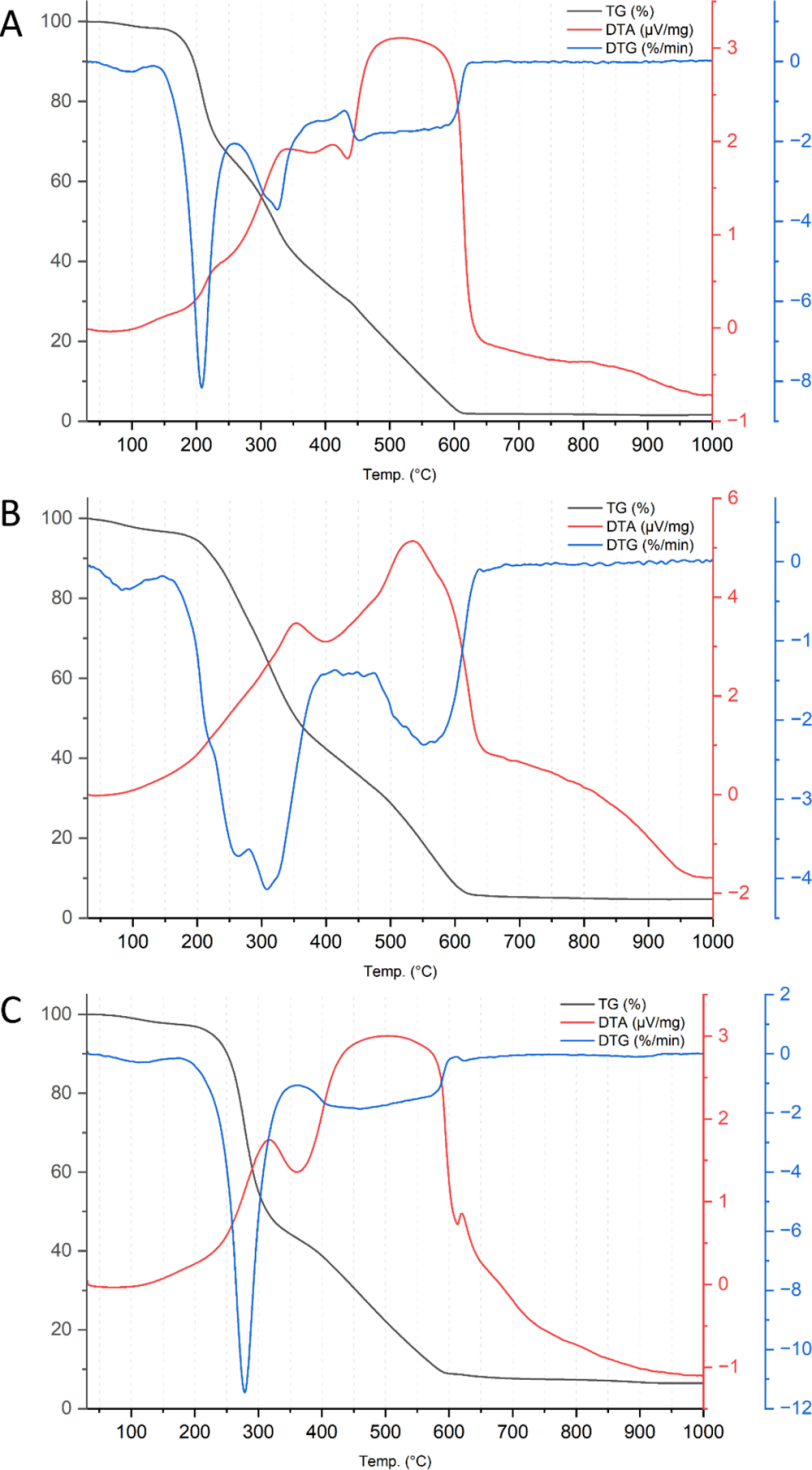
Thermal curves recorded for apple pomace (A), rapeseed pomace (B), and potato peelings (C): TG, DTA, and DTG curves (air flow, heating rate of 10 K/min).

Based on the thermal analysis, it was found that CDs are formed from monosaccharide-rich materials at >150 °C during thermal decomposition, with monosaccharides, pectins, proteins, and hemicellulose being the main sources. Therefore, it was concluded that the most favorable process temperature should be around 200 °C, where a rapid endothermic transformation occurs. The maximum process temperature was determined to be 220 °C. As a result of the trial processes, temperatures were chosen for the synthesis processes: 180 °C (*d_m_* = 24.8% ± 0.51%), 200 °C (*d_m_* = 29.4% ± 0.54%), and 220 °C (*d_m_* = 32.6% ± 0.83%).

The graph of the thermal analysis of the rapeseed pomace sample is shown in Figure 2B. An endothermic transformation peak is observed with a maximum at about 80 °C, associated with the evaporation of moisture and light volatiles from the material. In the range of about 150–350 °C, the first stage of material degradation resulting from 3 overlapping transformations can be observed, leading to a total mass loss of approximately 50%, with a relatively rapid mass loss, with the first transformation visible as an inflection on the peak of the second transformation at about 210 °C with a maximum mass loss of about 2.3%/min, a second transformation with a mass loss maximum of about 3.7%/min at 260 °C and a third transformation with a maximum of about 4.1%/min at 310 °C. In the DTA curve, it can be observed that the transformation is exothermic; the overlapping transformations are identified successively with the decomposition of mono- and simple polysaccharides and the degradation of hemicellulose and cellulose. Chen and his team studied the decomposition of rapeseed pomace in a synthetic air atmosphere at a heating rate of 10 K/min. An analogous transformation was identified by them as being related to the evaporation and combustion of carbon-containing substances.^41^ Ucar and Ozkan studied the degradation of rapeseed pomace in an inert gas atmosphere at a heating rate of 5 °C/min. They described the analogous stage of mass loss as mainly related to the degradation of hemicellulose with the release of gaseous products, along with the degradation of cellulose and lignin.^42^ In a further stage of the process, in the range of about 350–475 °C, the transformations occurring lead to a weight loss of up to approximately 30% of the initial weight, and the rate of weight loss stabilizes at about 1.4%/min. Ucar and Ozkan determined that this stage is mainly related to the further degradation of cellulose and lignin present in the marc, resulting in the release of gaseous products and the formation of bio-oil and biocarbon.^42^ Smets and his team conducted a thermal analysis of rapeseed pomace to select temperatures for the flash pyrolysis process. They determined that in this step, besides the decomposition of cellulose and lignin, the evaporation of triglycerides can also occur along with their decomposition into fatty acids.^43^ Subsequently, it is possible to distinguish another stage, a slow mass decrease, in the range of about 475–635 °C, the sample at the end of the stage reaches a mass of about 5.5% of the initial mass, which is associated with an exothermic transformation with a maximum mass loss rate of about 2.3%/min at about 530 °C, This is a transformation associated with the combustion of the remaining sample, but also with the decomposition of the remaining lignin. Chen et al. described this step as being directly related to the combustion of previously formed char.^41^ In the thermal analysis graphs presented by Ucar and Ozkan, this peak has a smooth course, so it can be concluded that, in addition to combustion, thermal decomposition is still taking place at this stage.^42^ At >635 °C, a final, slowed exothermic transformation takes place, resulting in the sample reaching a constant mass of approximately 4.7% of the initial mass.

Based on the thermal analysis, it was determined that the formation of CDs would mainly take place in the first stage of sample degradation in the range of about 200–300 °C. Due to the high content of proteins and monosaccharides, which degrade at the initial stage of the process, the maximum temperature was set at 300 °C; after trial runs, the maximum temperature was lowered to 260 °C. Temperatures were selected for the synthesis processes: 220 °C (*d_m_* = 13.3% ± 0.45%), 240 °C (*d_m_* = 20.3% ± 0.73%), and 260 °C (*d_m_* = 25.7% ± 0.73%).

Figure 2C shows a graph of thermal analysis of a sample of potato peelings, where the presence of an endothermic transformation is observed with a maximum at about 115 °C, associated with the evaporation of moisture and volatiles from the sample. Then, in the 200–320 °C range, there is a peak associated with the first stage of decomposition, which results in a weight loss of approximately 50% of the sample with a maximum weight loss rate of about 11.5%/min at 280 °C, which was identified as related to the endothermic transformation. Liang and McDonald studied potato peeling in an inert gas atmosphere with varying heating rates. They determined that this stage was mainly associated with the breakdown of hemicellulose and starch and the onset of cellulose, lignin, and suberin breakdown.^31^ In the range of about 320–405 °C, another stage of transformation can be observed, with a mass loss of 10% in this stage. The mass loss rate in this stage decreases to 1%/min. A sharp peak associated with endothermic transformation, thermal depolymerization, appears on the DTA curve. Based on Liang and McDonald’s study, it was concluded that this stage is associated with further decomposition of cellulose, lignin, and suberin.^31^ In a further stage of the process in the range of about 405–590 °C, the material at the end of the stage reaches approximately 9% of the initial mass, the rate of mass loss stabilizes at about 1.75%/min, and an exothermic transformation occurs. This stage is associated with the combustion of the material and carbon formed in the previous stages and further decomposition of lignin, among other things. In the temperature range of 590–620 °C, there is evidence of another endothermic transformation, evident by a peak at 612 °C, accompanied by no discernible change in the sample’s mass. At >620 °C, the final stage of the thermal process occurs, and the sample reaches a constant mass of about 6.5% of the initial mass.

Based on the thermal analysis, it was determined that the formation of CDs begins in the first stage of degradation (200–320 °C), with degradation mainly of hemicellulose and starch, and because of the rapid course of further mass loss and the high mass loss obtained, the maximum process temperature was set as 260 °C. For the synthesis processes, temperatures were chosen: 220 °C (*d_m_* = 8.5% ± 0.86%), 240 °C (*d_m_* = 25.1% ± 1.47%), and 260 °C (*d_m_* = 40.9% ± 0.89%).

### 3.3 Spectroscopic Analysis of Substrates and Products Obtained after the Thermal Process

Table 4 summarizes the characteristic peaks appearing in the FT-IR spectra of substrates and products after the thermal process, along with their changes in the process and likely origin.

**Table 4:** Summary of FT-IR Absorption Band Values in Feedstock and Products after Thermal Treatment

material	wavenumber [cm^–1^]	thermal behavior	functional group (type of vibration)	assignment	lit.
all	3350	weakening	O–H stretching, inter- and intramolecular hydrogen bonds	polysaccharides	^45^,^46^
R	3010	unchanged	cis C–H stretching, in alkenes	oil residuals	^47^
R	2960	unchanged	asymmetric C–H stretching, probably in CH_3_ groups	branched alkenes	^48^
J, Z	2920	unchanged	asymmetric C–H stretching, probably in CH_2_ groups	polysaccharides,	^45^
R	2920	unchanged	asymmetric C–H stretching, probably in CH_2_ groups	polysaccharides, lipids	^49^
J, Z	2850	unchanged	symmetric C–H stretching probably in CH_2_ groups	polysaccharides	^45^
R	2850	unchanged	symmetric C–H stretching probably in methylene CH_2_	polysaccharides, lipids	^49^
R	1740	strengthening	stretching vibration of esters C=O groups	oil residuals	^47^,^50^
J	1730 → 1705	shift, strengthening	stretching vibration of C=O groups: in initial position in ketones/aldehydes/esters, after 1st shift unconjugated ketones/aldehydes	hemicellulose, cellulose	^45^,^46^
Z		shift, strengthening			
R	1710	strengthening	carboxylic C=O stretching	oil residuals	^47^
J	1640	weakening (decay)	C–O bending associated with H–O–H deformation	water adsorbed in polysaccharides structure (in amorphous regions)	^51^,^52^
Z	1650				
R	1645 → 1655	shift, weakening	amide I vibration −N–H stretching, C–O bending associated with H–O–H deformation	proteins, lignin	^47^,^50^,^53^
J	1610 → 1605	shift, unchanged	vibrations of the aromatic ring C=C and overlapping C–O stretching vibrations	polysaccharides (lignin)	^45^,^54^,^55^
R	1590	strengthening			
Z	1600 → 1585	shift, strengthening			
R	1540	weakening	amide II band–N–H bending	proteins	^47^,^49^,^50^,^53^
J	1520 → 1515	shift, strengthening	CH_2_ stretching vibrations in aromatic ring	polysaccharides (lignin)	^54^,^55^
R	1515	unchanged			
Z	1515	unchanged			
J	1440	weakening	scissor vibrations of the CH_2_ groups	polysaccharides (cellulose, starch)	^52^
Z	1445	strengthening			
R	1455	unchanged	scissor vibrations of the CH_2_ groups, amide III band N–H bending	polysaccharides (cellulose, starch) and proteins	^47^,^53^
J	1415	weakening (decay)	CH_2_ bending, −COO stretching	polysaccharides (starch)	^52^,^56^
R					
Z					
J	1370	weakening (decay)	bending vibrations of C–H and CH_2_, symmetric C–H bending from methoxy (−OCH_3_) group	polysaccharides (cellulose, hemicellulose)	^45^,^46^,^52^,^55^
R	1375	strengthening			
Z	1370	strengthening			
J	1240	weakening (decay)	vibrations of C–O–C, C–C stretching, C–O stretching, C–H bending, O–H plane deformation (−COOH)	polysaccharides	^46^,^54^
R	1235	weakening			
Z	1235	weakening			
J	1200	unchanged	O–H bending	polysaccharides	^45^
Z		weakening (decay)			
J	1145	weakening (decay)	C–O–C asymmetrical stretching, aromatic C–H in plane deformation, glucose ring vibrations	glycosidic bond and the glycosidic ring in pyranose form	^45^,^46^,^55–57^
R	1155	unchanged			
Z	1150	weakening			
J	1100	weakening (decay)	C–H in ring asymmetric stretching, C–C and C–O stretching	polysaccharides	^46^,^58^
R	1095	weakening			
Z	1100	weakening			
Z	1045	weakening (decay)	C–C stretching, C–O stretching, C–O–H bending	crystalline region of polysaccharides (cellulose and starch), glycosidic ring in pyranose form	^56–58^
	1075	weakening			
all	1020–1030	weakening	C–O, C=C and C–C–O stretching	amorphous region of polysaccharides (cellulose and starch), glycosidic ring in pyranose form	^45^,^56^,^57^
R	990	weakening (decay)	C–H bending out of the plane	branched alkenes	^48^
J	925	weakening (decay)	skeletal vibrations of α-1,4-glycosidic bonds in pyran ring	glucose subunits in polysaccharides	^45^,^51^,^56^,^57^
R	930				
Z	925				
J	865	weakening (decay)	skeletal vibrations of α-1,4-glycosidic bonds, C–H out of plane vibrations in α-configuration	glucose subunits in polysaccharides	^45^,^46^,^51^,^56^,^57^
Z	850				
J	820	weakening	C–H bending out of the plane	possible substituted polycyclic compounds	^48^
J	775 → 795	shift	C–C stretching	glucose subunits in polysaccharides	^51^,^56^
Z	760 → 780				
R	720	weakening	N–H bending out of plane	proteins	^48^
J	585	weakening	C–C–O, C–O–C, C–C–C deformation vibrations	pyranose rings	^56^,^59^
Z	575				
J	515	unchanged	C–C–O, C–O–C, C–C–C deformation vibrations	pyranose rings	^59^
R	520				
Z	520				

The spectra of apple pomace (J) and the samples derived from it after the thermal process (J-···-10) are summarized in Figure S6, the spectra of rapeseed pomace (R) and the products of the thermal process derived from it are summarized in Figure S7, the spectra of potato peelings (Z) along with the products of the thermal process at t = 10 min (Z-··· −10) in Figure S8 and potato peelings (Z) along with the products of the thermal process with *t* = 30 min (Z-···-30) are summarized in Figure S9.

The bands common to all spectra are those associated with the presence of hydrogen bonds (3350 cm^–1^), bands identified as characteristic for polysaccharides, mainly cellulose, hemicellulose and lignin (2920, 2850, 1600, 1515, 1370, 1240, 1100, 1030 cm^–1^) and bands associated with the presence of glycosidic rings in the form of pyranose (1150, 1030, 925, 520 cm^–1^). During the thermal process, it can be observed, common to all raw materials, the weakening of bands at approximately 3350 and 1645–1650 cm^–1^. It can be associated with the loss of adsorbed and bound water. The bands associated with the presence of lignin do not change significantly at the temperatures studied. The weakening of bands at approximately 1440–1455, 1370, 1235, 1200, 1150, and 1100 cm^–1^ can be identified with the decomposition of polysaccharides, with the breaking of C–C and C–O bonds being observed. Moreover, the bands identified with the presence of pyranoses are weakened, so it can be inferred that the decomposition of pyranose rings occurs into short keto acids.^44^

The spectra exhibit analogous changes upon thermal processing of both apple pomace and potato peel. The band at 1730 cm^–1^ undergoes a shift and enhancement, which can be associated with the probable thermolysis of polysaccharides (including cellulose) with the disruption of the glycosidic bond and thus the transformation of the C–O–C grouping to C=O, this transformation may also be the cause of the weakening and consequent disappearance of the band at 1630 and 1200 cm^–1^.

For rapeseed pomace, on the spectra, in addition to the bands associated with the presence of polysaccharides and pyranoses, the appearance of new bands at 3010, 2960, 1740, 1710, 1645, 1540, 1455 cm^–1^ can also be observed, as well as a significant strengthening relative to the other raw materials of the bands at 2920, 2850 cm^–1^, which can be linked to the presence of proteins rich in N–H groups and oil residues after pressing, rich in unsaturated bonds. The bands at 1740 and 1710 cm^–1^ undergo strengthening in the thermal process, when the band at 1645 cm^–1^ weakens until it disappears, which can be explained analogously to that of the other raw materials.

### 3.4 Morphology of Biomass-Derived CDs

Table 5 summarizes the characteristic peaks appearing in the FT-IR spectra (Figure 3) of dried CDs obtained from different substrates at varying temperatures and the probable origin of the listed bands.

**Figure 3:**
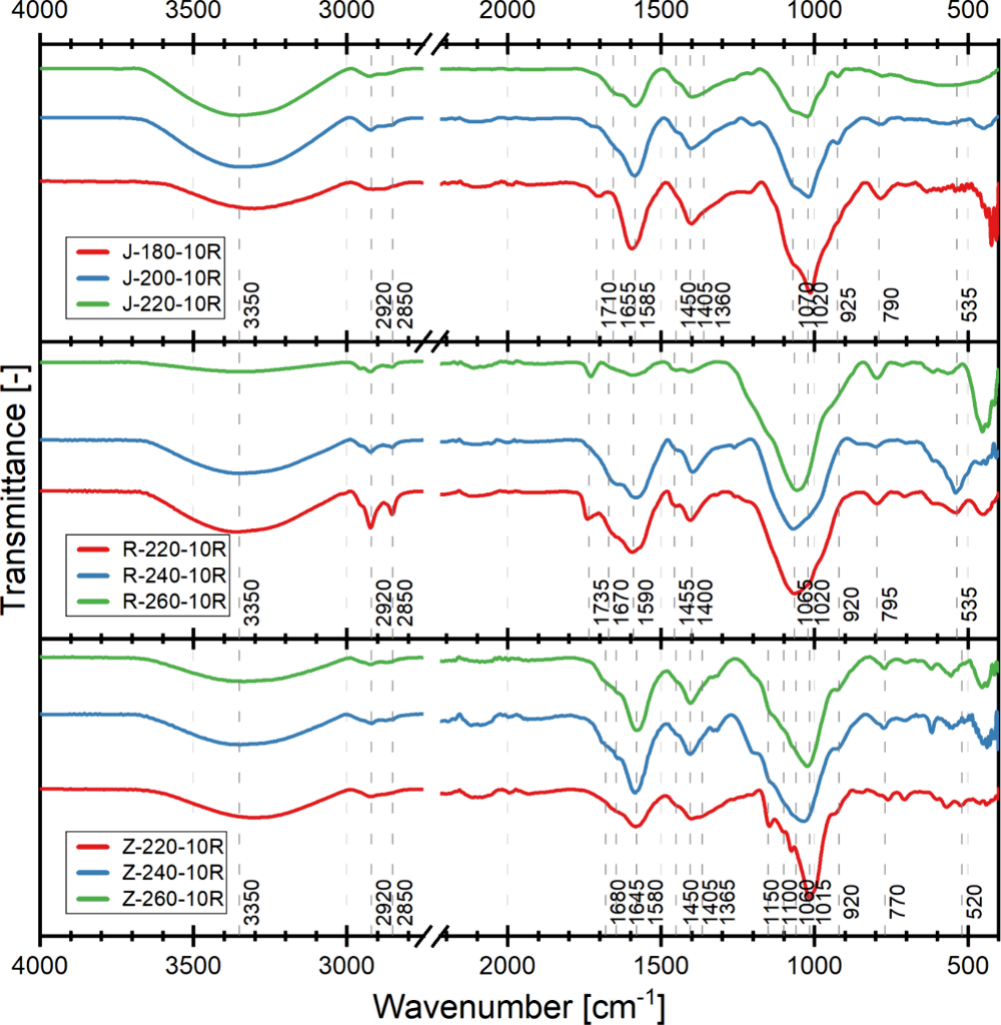
FT-IR spectra of CDs obtained from the biowaste feedstock: apple pomace (J), rapeseed pomace (R), and potato peelings (Z).

**Table 5:** Summary of FT-IR Absorption Band Values in CDs from Apple Pomace (J), Rapeseed Pomace (R), Potato Peelings (Z)

sample	wavenumber [cm^–1^]	functional group (type of vibration)	assignment	lit.
all	3350	O–H stretching, inter- and intramolecular hydrogen bonds	saccharide residue, CDs	^45^,^46^
all	2920	asymmetric C–H stretching, probably in CH_2_ groups	saccharide residue, aromatic structures	^45^
all	2850	symmetric C–H stretching probably in CH_2_ groups	saccharide residue, aromatic structures	^45^
R-···-10R	1735	stretching vibration of C=O groups	saccharide and lipids residue, CDs	^60^
J-···-10R	1705	stretching vibration of C=O groups	saccharide residue, CDs	^60^
Z-···-10R	1680	stretching vibration of C=O groups	saccharide residue, CDs	^60^
J-···-10R	1645–1655	vibrations of the aromatic ring −C=C– and overlapping C–O stretching vibrations	CDs	^61^
Z-···-10R				
all	1580–1590	–COO asymmetrical stretching	CDs	^60^
all	1445–1455	scissor vibrations of the CH_2_ groups, amide III band N–H bending	saccharide and proteins residue, CDs	^47^,^52^
R-···-10R	1400–1405	–CH_2_ bending, −COO symmetrical stretching	saccharide residue, CDs	^60^
Z-···-10R				
J-···-10R	1365–1370	bending vibrations of C–H and CH_2_, symmetric C–H bending from methoxy (−OCH_3_) group	saccharide residue, CDs	^45^,^55^
Z-···-10R				
Z-···-10R	1150	C–O–C asymmetrical stretching, aromatic C–H in plane deformation, glucose ring vibrations	saccharide residue (pyranose form), CDs	^45^,^46^,^55–57^
Z-···-10R	1100	C–H in ring asymmetric stretching, C–C and C–O stretching	saccharide residue, CDs	^46^,^58^
all	1060–1070	C–C stretching, C–O stretching, C–O–H bending	saccharide residue (pyranose form), CDs	^56–58^
all	1015–1020	C–O, C=C and C–C–O stretching	saccharide residue (pyranose form), CDs	^45^,^56^,^57^
all	920–925	skeletal vibrations of α-1,4-glycosidic bonds in pyran ring	glucose residues	^45^,^46^,^51^,^56^,^57^
all	770–795	C–C stretch	glucose residues, aromatic structures	^51^,^56^,^61^
all	520–535	C–C–O, C–O–C, C–C–C deformation vibrations	pyranose rings	^59^

All the CDs spectra have peaks at 3350, 2920, 2850, 1580–1590, 1445–1455, 1060–1070, 1015–1020, 920–925, 770–795, 520–535 cm^–1^, and these are bands largely in common with those found in waste and products of their thermal degradation. The presence of common functional groups in polysaccharides and CDs, particularly oxygen-rich groups, along with water-soluble saccharide residues, may contribute to the observed bands associated with pyranoses.

In the case of apple pomace CDs, as the temperature of the synthesis process increases, a decrease in the intensity of the bands can be observed at 1705 and 1020 cm^–1^. These bands are associated with carbonyl C=O and alkoxy C–O groups. Also, an increase at 1655 cm^–1^, a band associated with the vibration of the aromatic ring, can be observed. Thus, it can be concluded that the use of a higher temperature (220 °C) promotes the formation of aromatic structures and, as a result, crystalline CDs. With CDs from rapeseed pomace, with an increase in process temperature, a decrease in the intensity of bands at 1735, 1590, 1455, 1020 cm^–1^ can be observed, while there is no increase in the intensity of bands associated with aromatic bonds. On the spectra of CDs from potato peelings, it is possible to observe a decrease in the intensity of bands at 1680 and 1365 cm^–1^ associated with C=O and OCH_3_ groups, respectively, and an enhancement of bands at 1645, 1580, 1405, and 1100 cm^–1^. These bands are associated with vibrations of aromatic rings and oxygen-rich functional groups. It can be concluded by analogy with apple pomace that the use of higher temperature (260 °C) promotes the formation of crystalline CDs.

Figure 4 compares TEM microphotographs to histograms of the size distributions obtained from the posted CDs microphotographs. TEM microphotographs from which crystal planes were determined are included in Figure S11. The CDs obtained by the J-220 process have a narrow distribution with an average size of 4.0 ± 0.59 nm and a *d*-spacing of 0.27 ± 0.011 nm. For R-240 CDs samples, the size distribution is uneven, with an average particle size of 11.2 ± 2.39 nm; due to the amorphous structure of CDs, no crystal planes were observed. Z-260 CDs samples show a narrow size distribution with an average size of 5.0 ± 1.15 nm; the presence of varying distances between *d*-spacing planes can be observed, being 0.22 ± 0.016 nm, 0.28 ± 0.008 nm, and 0.31 ± 0.016 nm.

**Figure 4:**
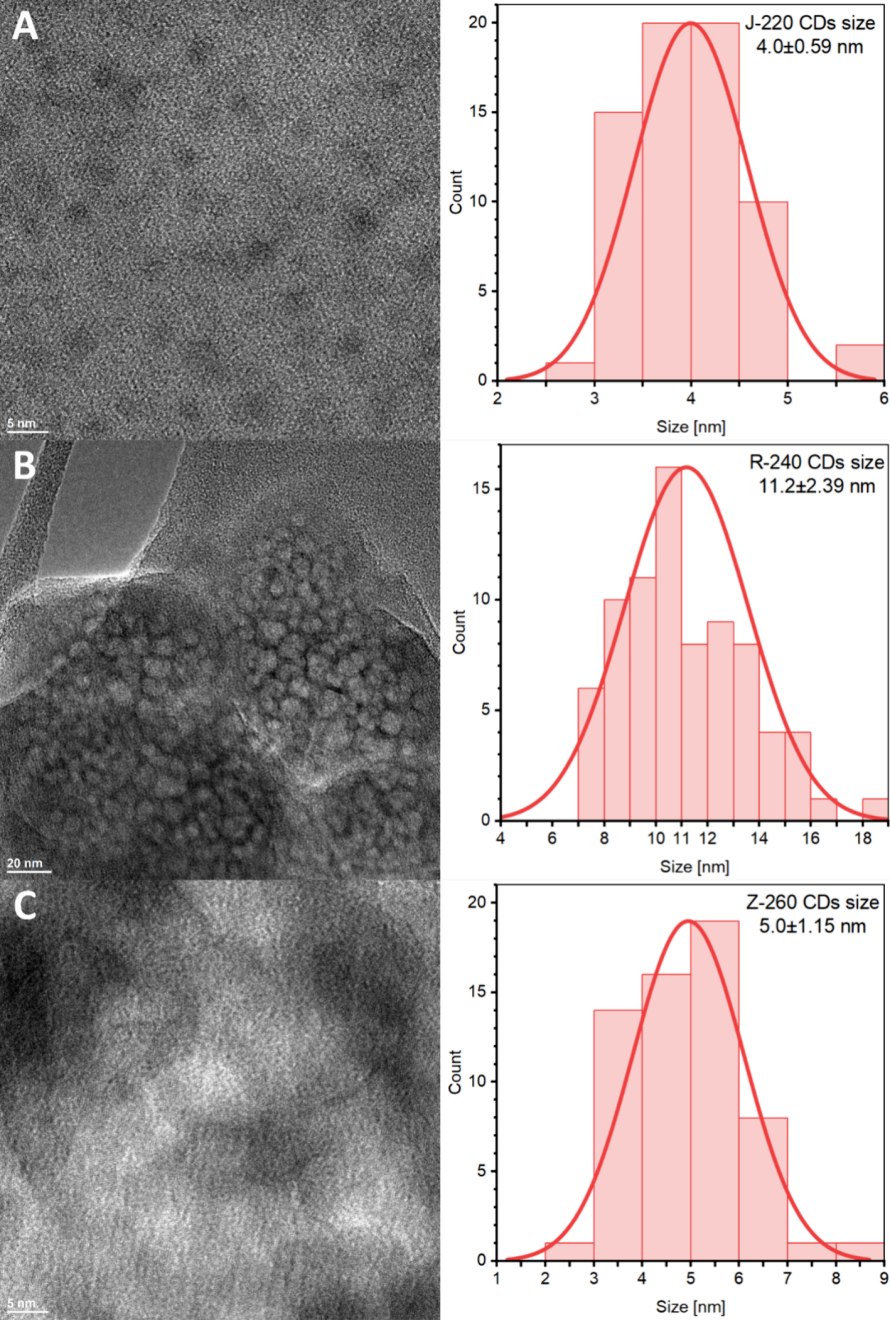
HR-TEM microphotographs of CDs obtained from the biowaste feedstock: apple pomace (A), rapeseed pomace, (B) and potato peelings (C), along with size histograms.

Figure 5 summarizes the results of XRD analyses of CDs samples formed by the J-220, R-240, Z-260 process (A), as well as diffractograms of J-220 (B) and Z-260 (C) samples subjected to deconvolution; full diffraction spectra subjected to deconvolution are included in Figure S12 for J-220 and Figure S13 for Z-260.

**Figure 5:**
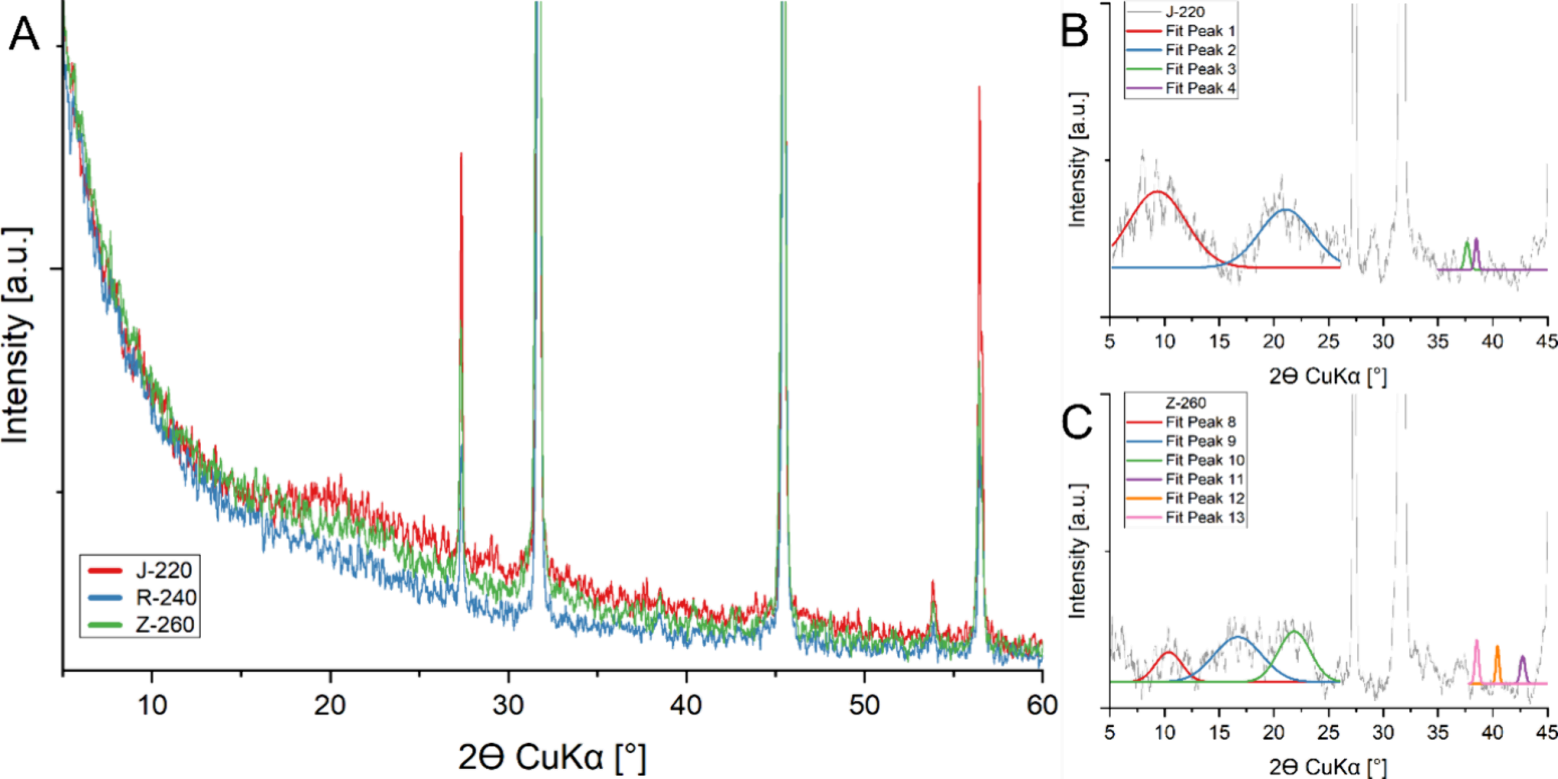
Raw X-ray diffractograms of dried CDs from J-220 apple pomace, R-240 rapeseed pomace, and Z-260 potato peelings (A) along with J-220 (B) and Z-260 (C) diffractograms after processing.

Peaks associated with the presence of residual NaCl are summarized in Table S2. Salt is residual after neutralization, and dialysis of the sample, which removes 99% of the ions present in the system after the neutralization process, is present in all spectra. The values of the 2θ angles along with the corresponding crystal planes and *d*-spacing values are summarized in Table 6.

**Table 6:** Values of Key 2θ Angle in CD Analysis, along with Reference and Determined d-Spacing Values

2θ range [°]	plane	structure	2θ (lit.) [°]	*d*-spacing (lit.) [nm]	lit.	sample	2θ value [°]	*d*-spacing [nm]
5–15°	001	graphene oxide	10.2	0.8	^62^	J-220	9.37	**0.94**
						Z-260	10.4	**0.85**
15–25°	110	cellulose	16.7	0.53	^63^	Z-260	16.72	**0.53**
	002	graphite	26.6	0.34	^64^	J-220	21.08	**0.42**
	200	cellulose	22.1	0.4	^63^	Z-260	21.89	**0.41**
35–45°	100, 001	graphite turbostratic carbon	43.5, 44.5	0.21, 0.2	^64^,^65^	J-220	37.65	**0.24**
							38.5	**0.23**
						Z-260	38.54	**0.23**
							40.43	**0.22**
							42.74	**0.21**

In the 5–50° range, crucial for CDs analysis, differences between the diffraction spectra of different samples can be observed. In the case of CDs obtained from rapeseed (R-240), no bands characteristic of CDs were observed, which indicates that the sample is amorphous and confirms the results of HR-TEM analysis. The band most characteristic of CDs has a peak at about 26° and a distance between the planes of about 0.34 nm. With the samples studied, the band present at about 15–25° is broad and vague, with a maximum at about 21.5°. This band can be associated with the presence of graphene structures with a crystal plane (002) with a small number of layers.^66^ A diffraction band with a peak at about 22.5° is also present in the crystal structure of cellulose and is associated with a crystal plane (200), with a distance between planes equal to 0.39 nm. With cellulose with an amorphous structure, a diffraction band can be observed at about 20.5.^67^ In the case of CDs from potato peelings, a peak was also observed at about 17°. An analogous diffraction band can also be observed in the spectra of crystalline cellulose, and it is associated with a crystal plane (110) with a *d*-spacing of 0.53 nm.^67^ There is also observed the presence of a small, broad, and fuzzy band from 5 to 15°, with a peak at about 10°; the *d*-spacing values determined in this range are 0.85–0.94 nm. This band can be identified with the crystal planes (001) present in the graphene oxide structure.^62^ There are also numerous weaker bands present between 35 and 45° and a band covered by a peak from NaCl at 45°, which are probably related to the (100) and (001) planes in the graphite structure.^64,68^ The band at around 35° is also characteristic of crystalline cellulose, corresponding to a lattice spacing of 0.22–0.24 nm.^63^

The *d*-spacing values determined from the diffractograms for CDs J-220 are close to those read from HR-TEM microphotographs (0.23–0.24 and 0.27 nm, respectively), similarly for CDs Z-260 (0.22–0.31 and 0.21–0.23 nm, respectively). Moreover, these values are very close to those in the literature for CDs. The bands associated with graphite in the XRD diffractograms are visibly blurred and shifted toward lower angles, probably due to the small number of layers and the size of the particles studied.^69^ The significant differences in the distances between the crystal planes may be related to the varying degrees of oxidation in the different CDs samples. It is known that a greater number of oxygen-containing functional groups on the outer edges of the particles is associated with an increase in the distances between the planes.^70^

### 3.5 Optical Properties of Biomass-Derived CDs

Absorbance spectra of CDs synthesized from waste biomass (Figure 6) are characterized by different trends relative to each other, but in a range consistent with other CDs reported in the literature obtained from different substrates.^71^

**Figure 6:**
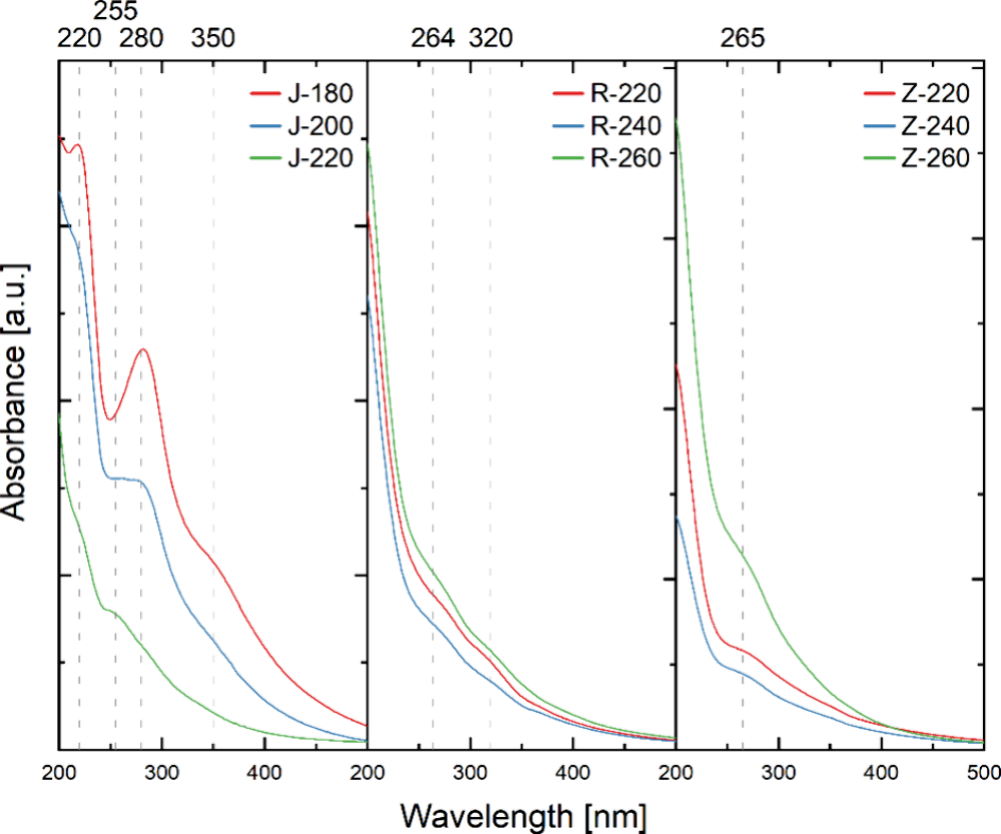
UV–vis spectra of obtained CDs in the range of 200–500 nm (in sequence left–right from apple pomace, rapeseed pomace, and potato peelings).

The spectrum of CDs achieved from apple pomace is characterized by the presence of two peaks in the middle UV range with maxima at 220 and 255–280 nm and a single mild peak in the near UV range at about 350 nm. With the CD spectra obtained from rapeseed pomace and potato peelings, the absorbance band at about 265 nm is common to them. In addition, a peak at about 364 nm is present in the CDs spectra from rapeseed pomace. In addition, on the spectra of CDs from apple pomace, as the synthesis temperature increases, a progressive shift can be observed in the peak present at about 220 nm toward shorter wavelengths, beyond the range studied, and from 280 to 255 nm. On the other CDs spectra derived from the wastes (rapeseed pomace and potato peelings), it can be observed that as the process temperature increases, there is a progressive increase in the mild absorbance band with a maximum at around 260 nm and a peak present outside the range studied, whose tail is observed at around 200–220 nm. The peaks present in the middle UV range are identified as probably related to π–π* interactions (C=C) in the carbon core with polyaromatic carbon structures and sp^2^ graphene-like carbon nanodomains of different sizes,^72,73^ based on this, it can be assumed that the increase in process temperature causes changes in the structure of the CDs core, probably related to the progressive graphitization of the carbon structures that make up the core. Absorbance bands in the near UV range are related to *n*–π* surface group interactions (C=O, C–OH, C–N),^72–74^ but usually associated with the middle UV absorption band π–π* interactions in the carbon core with sp^2^ hybridization are also an important component of this band. Additionally, interlayer charge transfers between graphene structures (in a single molecule or between multiple molecules) are another overlooked component of the interactions in this band.^75^

All fluorescence spectra gained at an excitation wavelength of λ_ex_ = 350 nm for CDs synthesized from waste biomass (Figure 7) are characterized by a blurred fluorescence band in the 400–600 nm range and 80–100 nm fwhm. The received emission spectra are characterized by a similar pattern, relative to the fluorescence spectra of CDs obtained from plant biomass by other researchers.^76,77^ All the evaluated samples have similar emission characteristics, with a mild sharpening of the peak in the J-180, R-220, R-240, and Z-260 samples in the range of about 420–440 nm.

**Figure 7:**
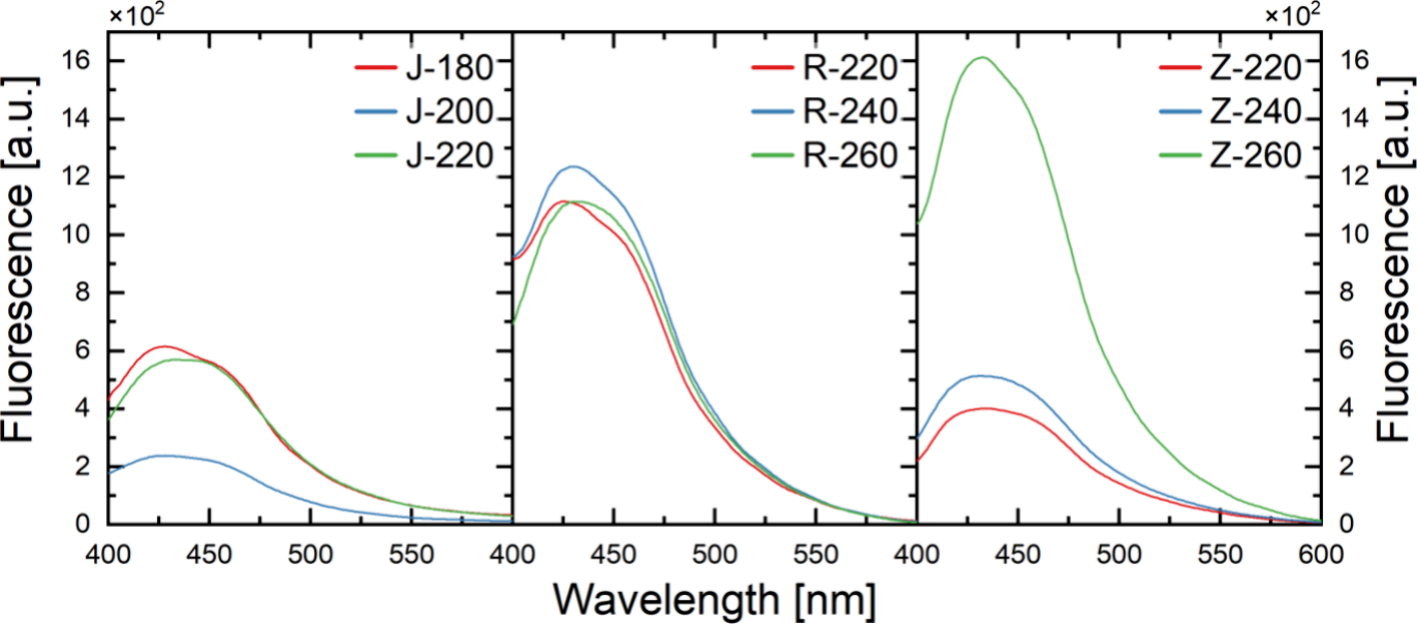
Fluorescence spectra with excitation by light with λ_ex_ = 350 nm of obtained CDs (in sequence left–right from apple pomace, rapeseed pomace, and potato peelings), at a fixed absorbance of <0,1 at λ_ex_.

The emission spectra of CDs from apple pomace are characterized by relatively low fluorescence, which is strongest for the product achieved in process at 180 °C (J-180), with an increase in synthesis temperature to 200 °C (J-200) there is a decrease in fluorescence, and then with a further increase in synthesis temperature to 220 °C (J-220) it increases to a level close to the fluorescence peak reached for the lowest temperature tested. With the fluorescence spectra of CDs obtained from rapeseed pomace at all synthesis temperatures studied, the emission intensity is at similar levels, for CDs achieved at 220 °C (R-220), as the process temperature increases to 240 °C (R-240), and the emission intensity increases slightly, followed by a decrease with the next increase in temperature (R-260), to values similar to those acquired for the lowest temperature. Observing the emission spectra of CDs from potato peelings, an increase in fluorescence intensity can be seen with an increase in process temperatures; the emission reached for a sample of CDs obtained at the highest temperature tested (Z-260) is clearly stronger than for samples synthesized at 220 °C (Z-220) and 240 °C (Z-240), moreover, it is the strongest among all the samples tested.

### 3.6 Yields of Biomass-Derived CDs

Table 7 shows the quantum yield values of prepared samples gained with λ_ex_ = 350 nm excitation and synthesis yields. These samples have fluorescence quantum yields of about 0–15% in the target visible light range (400–600 nm). There is no concentration quenching of fluorescence in the solutions of the obtained CDs. After irradiating selected samples with a 360 nm lamp for one hour, the fluorescence quantum yield drops by about 28 ± 2.7%. In the literature on CDs synthesized from biomass of various origins, quantum yields vary widely, from values of about 0.5% to as high as 59%.^78,79^ The image (Figure 8) shows the coloration of selected CDs suspensions along with the water shown as a reference.

**Table 7:** Fluorescence Quantum (λ_ex_ = 350 nm) and Synthesis Yields of CD Samples Obtained from Biomass Waste

sample	QY (%)	SY (%)	sample	QY (%)	SY (%)
J-180	0.0	47.6	R-220	0.8	15.4
J-200	4.3	10.8	**R-240**	**2.4**	**12.8**
**J-220**	**4.4**	**10.0**	R-260	10.4	16.3
Z-220	0.6	18.3	Z-220–30	0.1	50.3
Z-240	12.9	10.8	Z-240–30	1.0	35.7
**Z-260**	**14.5**	**12.3**	Z-260–30	2.4	28.4
Z-280–10	4.9	7.1	Z-280–30	1.3	25.2
Z-300–10	9.8	7.9	Z-300–30	11.8	8.2
Z-320–10	3.9	10.2	Z-320–30	6.3	10.0
Z-340–10	7.1	11.8	Z-340–30	8.7	9.1

**Figure 8:**
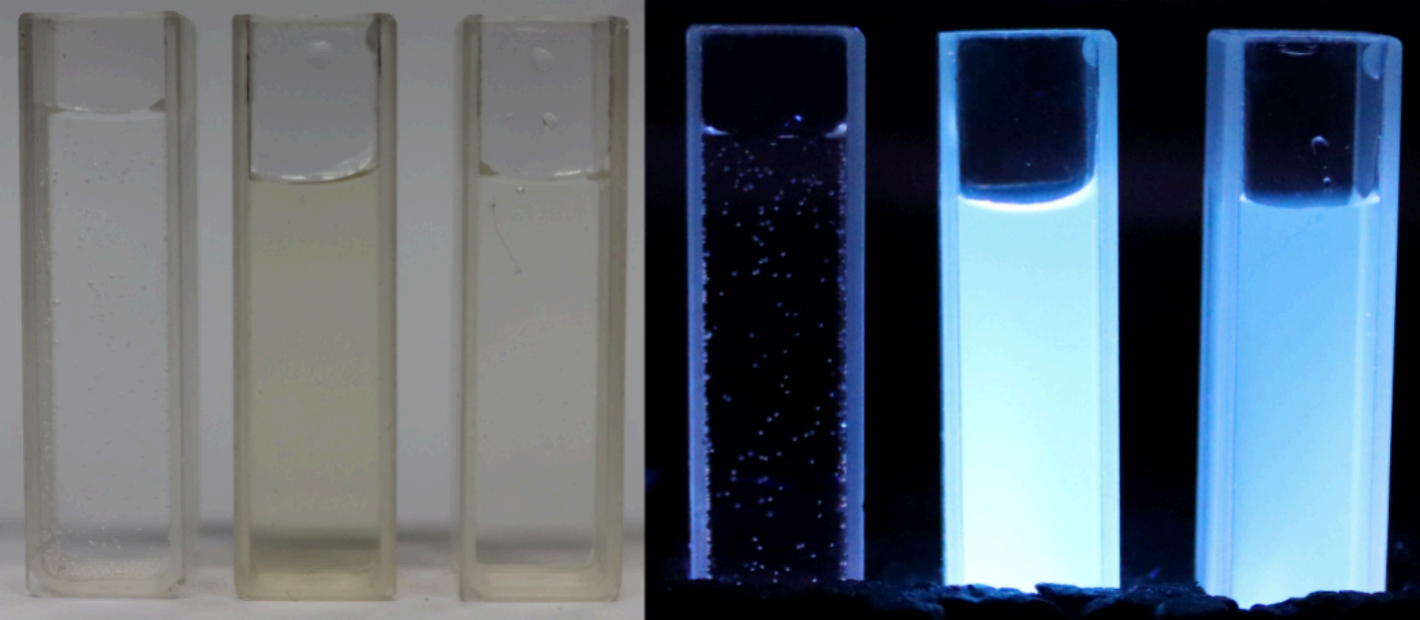
Sequentially water and CD suspensions of R-240 and Z-260 under visible (left) and UV light (right, λ_ex_ = 360 nm).

### 3.7 Optical Detection

Figure 9 presents the results of fluorescence area measurements as a function of the pH of the tested solution. An inset displays the same results with a narrowed axis. It can be observed that within the pH range 3–7, the fluorescence intensity changes only slightly. At extreme pH values, the fluorescence decreases by 10–15%. The most optimal pH range for measurement is 5–7.

**Figure 9:**
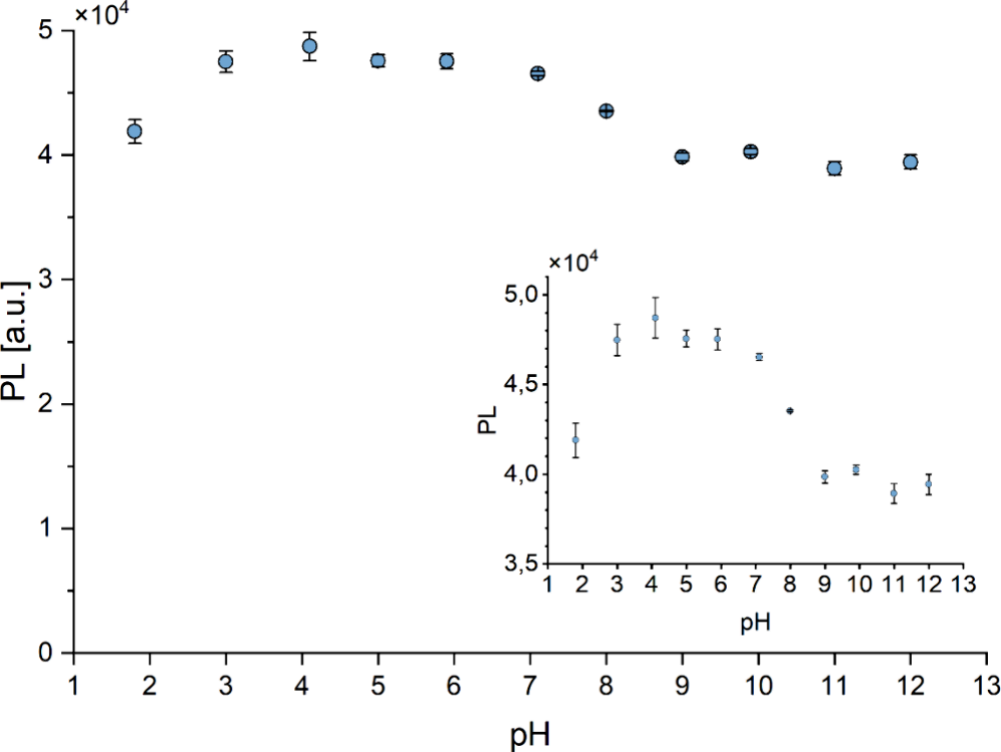
PL intensity of the CD solution in the 2–12 pH range.

Geng, studying the effect of pH on the fluorescence intensity of sulfur and boron-doped GCDs, observed that it remains approximately constant in the range of 6–8; thus, pH 7 was chosen for detection.^80^ Hashemi and Mousazadeh investigated the effect of the pH on the fluorescence of nitrogen-doped CDs. They observed significant differences in fluorescence intensity, with the strongest fluorescence at pH 6.^81^ In summary, the CDs obtained in this study exhibit a broader pH range suitable for detection due to their slight changes in fluorescence intensity. This phenomenon is particularly advantageous in the optical detection of compounds added in solution form, as it allows for the neglect of pH influence and simplifies the measurement process by eliminating the need for strict pH control.

Figure 10A summarizes the results of preliminary studies on fluorescence quenching by selected metal ions. Strong fluorescence quenching was observed for Fe^3+^ ions, of about 83%, compared with the fluorescence of the pure CDs solution. Pb^2+^ ions caused a decrease in the fluorescence of about 50%. Fluorescence extinction of about 20–30% was observed for Co^2+^, Cr^2+^, Cu^2+^, and Fe^2+^ ions. Figure 10B shows the results of detailed measurements of the dependence of fluorescence on the Fe^3+^ ion content. Results above the concentration of 2.5 mM (140 mg/L) are not shown on the graph, as they were excluded from the linear trend analysis due to significant deviation from the linear range. The range of concentration linearity was defined as 0.0125–1.25 mM.

**Figure 10:**
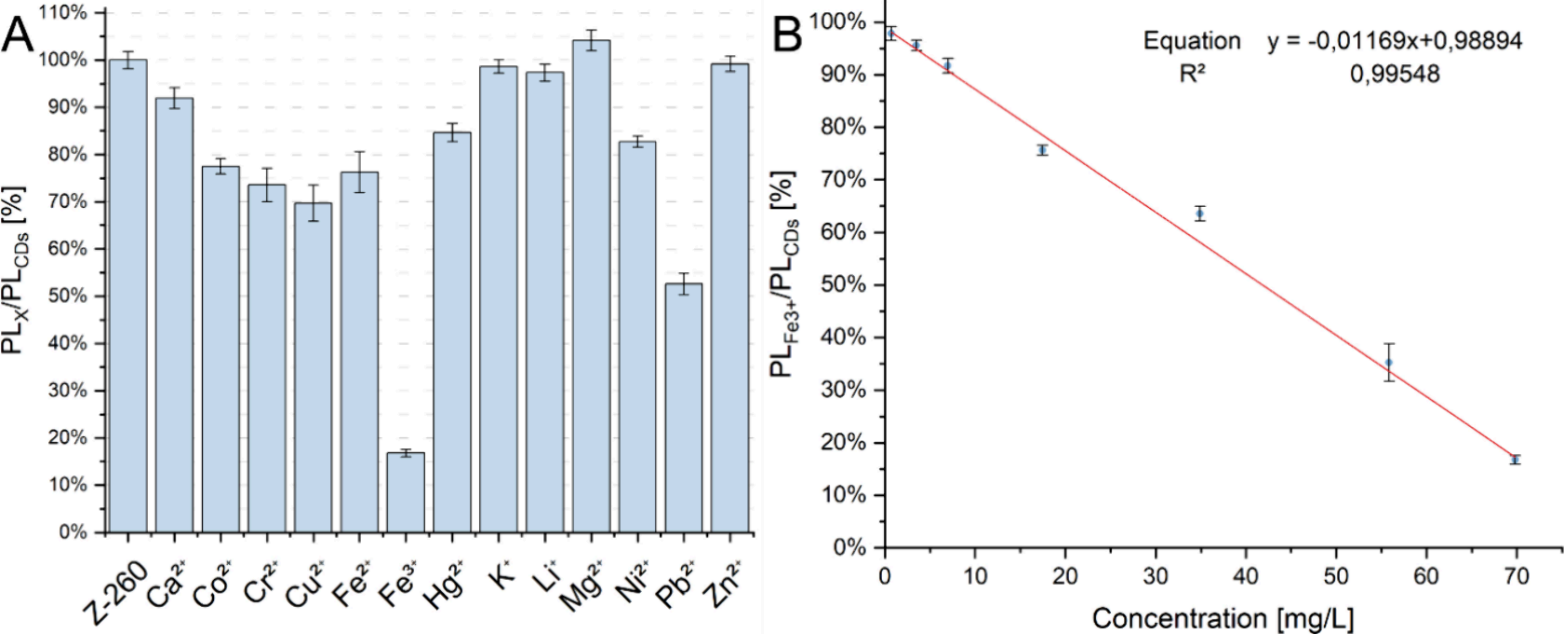
PL intensity of the CDs solution in the presence of various metal ions at a concentration of 1.25 mM (A) along with a calibration curve for the determination of Fe^3+^ ions in solution (B).

Figure 11A summarizes the results of preliminary studies on fluorescence quenching by water pollutants. For most of the samples, no fluorescence quenching was observed. Only for ibuprofen, weak fluorescence quenching was observed. Figure 11B shows the results of measurements of the dependence of fluorescence for a wider ibuprofen concentration range. Results above the concentration of 5 mg/L are not shown on the graph. The range of concentration linearity was defined as 0.25–5 mM.

**Figure 11:**
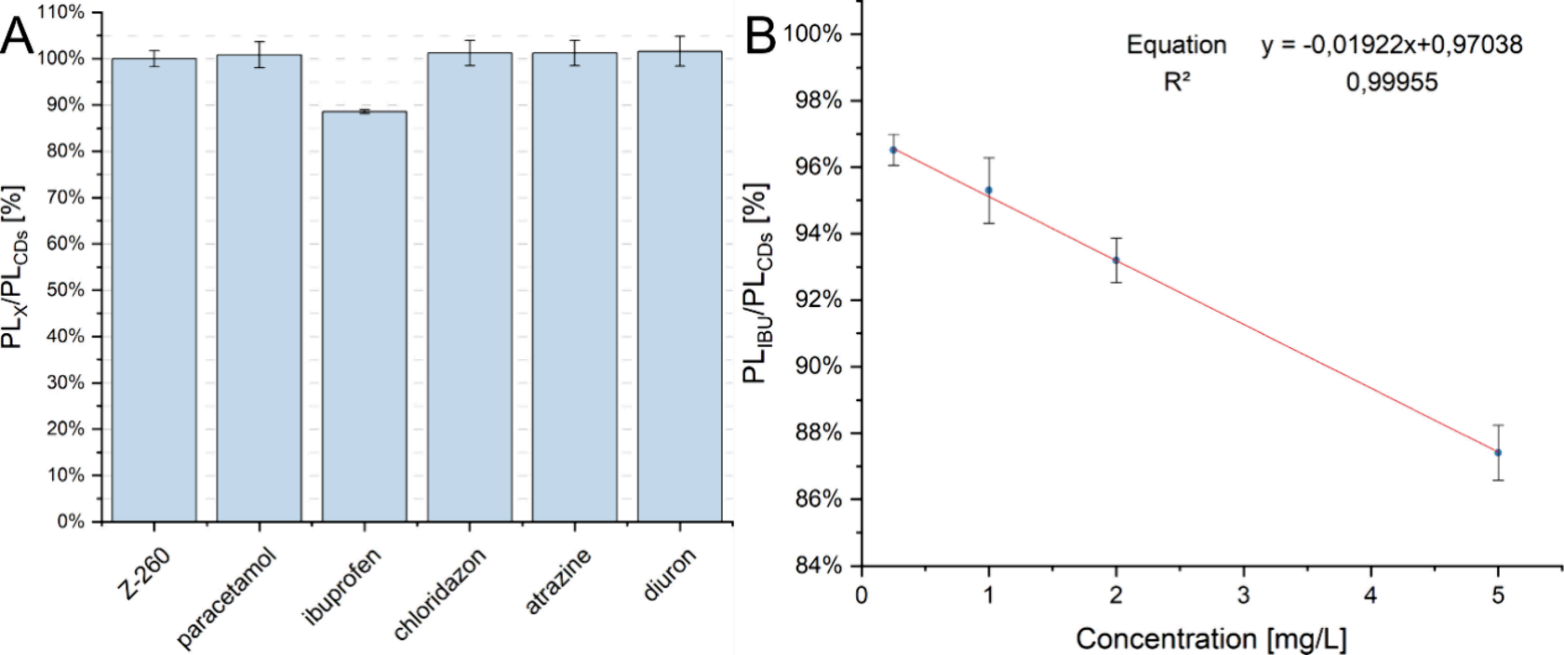
PL intensity of the CDs solution in the presence of various water pollutants at a concentration of 20 mg/L (A) along with a calibration curve for the determination of ibuprofen (IBU) in solution (B).

Table 8 summarizes the data describing the CDs obtained in this study and other studies covering the synthesis of CDs from waste materials of plant origin. It can be observed that most of the synthesis methods involve solvent-based methods, mainly hydrothermal and microwave-mediated synthesis.

**Table 8:** Comparison of the Optical Detection Parameters of the Obtained CDs with Literature Data

sensor	surface modification	detection method	analyte	linearity range	LOD	lit.
CDs		fluorescent	Fe^3+^	12.5–1250 μM	0.1244 mg·L	this study
			IBU	0.25–5 mM	0.0757 mg/L	
N-CDs	N-doped	fluorescent	Fe^3+^	5–60 μM	1.9 μM	^82^
CDs		fluorescent	Fe^3+^	0–400 μM	42,8 nM	^83^
N-CDs	N-doped	fluorescent	Fe^3+^	0.002–8 μM	13.8 nM	^81^
N-CDs	N-doped	fluorescent	Fe^3+^	0.1–1.3 μM	66 nM	^84^
CDs		fluorescent	Fe^3+^	0–0.5 mM	N/A	^85^
CDs	fluorescein	colorimetric	IBU	3–24 mM	0.1927 μg/L	^86^
CDs	polydopamine	fluorescent	IBU	nonlinear	1.58e–5 μM	^87^
BS-GQDs	B,S-doped	fluorescent	IBU	0.1–500 μM	30 nM	^80^

The table summarizes the values related to the optical detection of Fe^3+^ ions and ibuprofen obtained in this study compared with the data present in the literature. It can be observed that modified CDs, either by heteroatom doping or by surface attachment of modifying compounds (e.g., fluorescein or polydopamine), have a higher potential for detection. The linear detection range for Fe^3+^ ions obtained in this study is wider than in other presented studies, but its lower limit is higher; moreover, the LOD value is significantly higher than in the other cited studies. The results obtained for the detection of ibuprofen are better than those of the colorimetric method using CDs described by Shafi et al.; however, the linearity range and its lower limit are worse than those of the methods proposed by Geng and Mohiuddin et al. and Mohiuddin et al.

## 4 Discussion

Table 9 summarizes the quantitative data describing the CDs obtained in this study and other studies covering the synthesis of CDs from waste materials of plant origin. It can be observed that most of the synthesis methods involve solvent-based methods, mainly hydrothermal and microwave-mediated synthesis.

**Table 9:** Comparison of the Parameters of the Obtained CDs with Literature Data

biomass substrate	synthesis method	*T* [°C]	*t*	size [nm]	SY [%]	QY [%]	Λ_ex_ [nm]	P*L*_max_ [nm]	avg. PL_fwhm_ [nm]	lit.
apple pomace	torrefaction	220	10 min	4.0	10.0	4.4	350	430	96	this study
rapeseed pomace	torrefaction	240	10 min	11.2	12.7	2.4	350	430	100	
		260			16.3	10.4			96	
potato peelings	torrefaction	240	10 min		10.8	12.9	350	430	92	
		260		5.0	12.3	14.5			85	
olive pomace	pyrolysis	600	60 min	2.8	10	3	360	460	100	^88^
orange pomace	microwave	150	10 min	7.5	N/A	54.3	360	448	120	^77^
apple pomace	catalytic HTC	200	2 h	3.4	19	N/A	337	427	120	^76^
grape pomace		200		3.9		N/A	360	455	120	^76^
mango peels	thermal process	300	2 h	3.0	N/A	8.5	360	425	110	^89^
citrus peels	reflux condenser	180	12 h	N/A	N/A	N/A	420	510	110	^90^
banana peels	HTC	200	24 h	5.0	N/A	20	355	429	75	^91^
pomelo skins	HTC	180	8 h	3.3	N/A	76.5	345	440	100	^92^
orange peel	HTC with ultrasonication	200	6 h	3.9	N/A	N/A	260	460	144	^93^
lemon peel	thermal carbonization, microwave	200	2 h	4.5	N/A	49.5	330	445	100	^94^
avocado peels	HTC	180	24 h	2.9	14.8	N/A	350	404	100	^95^
banana peels	HTC	200	24 h	2.1	N/A	14.6	360	440	150	^96^
orange peels	thermal, HTC	180	12 h	N/A	N/A	18.0	360	440	150	^96^
orange peels	HTC	180	3 h	4.0	N/A	35.4	370	445	125	^97^
Jengkol peels	HTC	200	7 h	3.9	N/A	24.0	370	500	100	^98^

Sawalha and his team studied the synthesis of CDs by pyrolysis using olive pomace purified through extraction from oil and impurities. The process was conducted for 60 min at 600 °C, after which the material was dissolved and oxidized with H_2_O_2_ solution. Obtained CDs were highly crystalline, with a size of 2.8 nm and a *d*-spacing of 0.21 nm. They acquire CDs showing an emission maximum at 460 nm when excited with light of λ_ex_ = 360 nm. The CDs they achieve show a quantum yield of 3%.^88^

Kundu et al. synthesized green-light-emitting CDs from orange pomace using a microwave method. The pomace was prepared by suspending it in water, grinding it, and then separating the solid residue from the resulting solution. The solution underwent microwave treatment at 300 W for 10 min, at a temperature of 150 °C. The size of orange pomace-derived CDs is about 7.5 nm, and there are no visible lattice fringes. The highest fluorescence intensity occurs when excited with light of λ_ex_ = 360 nm, with a maximum of 448 nm, with a broad, tailing peak with an fwhm of about 120 nm. The quantum yield of the CDs they achieve is 54.26%.^77^

Ahmed, with a team, studied the synthesis of CDs from dried and crushed apple pomace and grape pomace by catalytic hydrothermal carbonization. The material was subjected to a thermal process at 200 °C for 2 h in the presence of KOH in ambient air, followed by water extraction of water-soluble substances from the resulting powder. The size of apple pomace-derived CDs is about 3.44 nm, with visible lattice fringes of 0.19 and 0.22 nm, while the grape pomace-derived CD size is 2.96 nm, with *d*-spacings of 0.19 and 0.21 nm. For CDs from apple pomace, the emission maximum occurs at excitation λ_ex_ = 337 nm, for CDs from grape pomace it occurs at λ_ex_ = 360 nm, and the emission peaks for both samples are broad (fwhm about 110–120 nm) and are in the range 380–600 nm. The quantum yield of fluorescence was not determined.^76^

Jiao et al. synthesized CDs from dried mango peels by a thermal process at 300 °C in ambient air for 2 h with subsequent oxidation with concentrated H_2_SO_4_. Obtained CDs were highly crystalline, with a size of 3 nm and a *d*-spacing of 0.22 and 0.42 nm. When excited with light of λ_ex_ = 310 nm, the fluorescence spectra exhibit emission maxima ranging from approximately 325–600 nm (fwhm of about 110 nm). The CDs they achieve have a quantum yield of 8.5%.^89^

Gudimella, with a team, studied the synthesis of CDs from dried citrus peels suspended in deionized water, in a 12 h process at 180 °C in a sand bath under a reflux condenser. Synthesized CDs were highly crystalline, with a size of 4.6 nm and a *d*-spacing of 0.2 nm. The emission maximum is observed when excited with light of λ_ex_ = 420 nm, reaching a peak with a maximum of 510 nm and an fwhm of about 110 nm. The quantum yield of fluorescence was not determined.^90^

Atchudan et al. synthesized CDs from banana peels, which were crushed in deionized water, and the resulting suspension was subjected to a hydrothermal process in an autoclave heated to 200 °C for 24 h. Obtained CDs were highly crystalline with a size of 5 nm and a *d*-spacing of 0.21 nm. The strongest fluorescence is observed at λ_ex_ = 355 nm with a relatively narrow emission peak at a maximum of 429 nm and an fwhm of about 75 nm. The CDs they achieve have a fluorescence quantum yield of 20%.^91^

Qi, with a team, studied the transformation of pomelo peel waste into nitrogen-doped CDs by a hydrothermal process in an autoclave. Dried and ground pomelo peels were mixed with ultrapure water and heated at 180 °C for 8 h, with no additional N modification. CDs from pomelo are characterized by an average size of 3.29 nm and probably an amorphous structure. The emission maximum is observed at 440 nm, when excited with light of λ_ex_ = 345 nm, the peak is characterized by a fwhm of about 100 nm. The quantum yield was determined as 76.47%.^92^

Murugesan, with a team, synthesized a nanosystem of CDs conjugated with Ag. For the synthesis of pure CDs from orange peel waste, they used combined hydrothermal carbonization at 200 °C for 6 h and ultrasonication for 50 min. Orange peel powder and citric acid were mixed with distilled water, and ammonia was added to maintain a neutral pH. The size of pure CDs is about 3.86 nm; there are no visible lattice fringes. The strongest fluorescence is observed at λ_ex_ = 260 nm with a relatively broad emission peak at a maximum of 460 nm and an fwhm of about 143.90 nm. The quantum yield was not determined.^93^

Kundu, Basu, and Maity studied the upcycling of Citrus limon peel waste into CDs in a two-stage process of thermal carbonization, followed by microwave irradiation. Lemon peels powder was heated in a muffle furnace at 200 °C for 2 h, after which the obtained powder was added to deionized water with 1 h of sonication, and subsequently transferred into a microwave reactor for 5 min (150 °C, 300 W). Obtained CDs were highly crystalline, with a size of 4.46 nm and a *d*-spacing of 0.36 nm. The emission maximum is observed at 445 nm; when excited with light of λ_ex_ = 330 nm, the peak is characterized by a fwhm of about 100 nm. The quantum yield was determined as 49.5%.^94^

Ferjani et al. synthesized CDs by hydrothermal carbonization of avocado peels. Powder of ground avocado peels was mixed with distilled water to form a paste, which was transferred into an autoclave and heated at 180 °C for 24h. The size of the synthesized CDs is about 2.9 nm with *d*-spacings of 0.21–0.23 nm. The strongest fluorescence is observed at λ_ex_ = 350 nm with an emission peak at a maximum of 404 nm and an fwhm of about 100 nm. The quantum yield was not reported.^95^

Patel et al. synthesized CDs from banana and orange peels in a hydrothermal reaction. Dried and ground banana peels were mixed with deionized water and ethanol, which was transferred into an autoclave and heated at 200 °C for 24 h. In the case of orange peels, the method was modified. Dried orange peels were carbonized in a hot air oven at 150 °C. The powder was washed with 0.1 M H_2_SO_4_, washed with deionized water, and dried at 150 °C for 2 h. The obtained powder was mixed with sodium hypochlorite for 4 h and then washed with water until the pH was attained. The resulting powder was mixed with water and placed in an autoclave at 180 °C for 12 h, and the obtained solution was washed with dichloromethane, centrifuged, and evaporated to gain CDs. The size of the synthesized CDs from banana peel CDs is about 2.1 nm, with no observed lattice fringes. For both synthesized CDs, the emission maximum is observed at 440 nm, when excited with light of λ_ex_ = 360 nm, the peak is characterized by a fwhm of about 150 nm. The quantum yield was determined as 18.0% for CDs from orange peels and 14.6% from banana peels.^96^

Han and the team studied the synthesis of CDs from waste fruit peel (banana peels, orange peels, dragon fruit peels). Dried materials were ground and suspended in water, and for nitrogen-modified CDs, they were mixed with ethylenediamine, next ultrasonicated for 30 min, and transferred to a reactor at 120–200 °C for 1–5 h. Only N-doped, orange-derived CDs were studied further. The size of the synthesized CDs is between 3.5 and 5.5 nm, with 0.215 nm lattice fringes. The strongest fluorescence is observed at λ_ex_ = 370 nm with an emission peak at a maximum of 445 nm and an fwhm of about 125 nm. The quantum yield was determined as 35.37%.^97^

Prayuo and co-workers synthesized nitrogen-doped CDs from jengkol peels. Dried peels were ground, the obtained powder was extracted by maceration in organic solvents, extract was transferred into an autoclave at 200 °C for 7 h. For the synthesis of N-doped CDs, ethylenediamine was added to extract and mixed until homogenization. Obtained CDs were highly crystalline. Pure CDs are characterized by a size of 3.927 nm and *a d*-spacing of 0.24 nm, while the N-doped CDs average size is 4.495 nm and the same *d*-spacing. The emission maximum is observed with excitation with light of λ_ex_ = 370 nm at 497 nm for pure CDs and 522 nm for N-doped CDs. The peaks are characterized by a fwhm of about 100 nm. The quantum yield was determined as 24% for pure CDs and 42% for N-doped CDs.^98^

It can be observed that a significant number of articles describe the synthesis of CDs from citrus fruit waste (peels, pomace), as well as other food industry waste, confirming the significant potential of this type of waste in the synthesis of carbon nanomaterials. Comparing the results with cited studies, it was found that the different origins and related differences in the composition of the substrates used in the synthesis of CDs have the greatest impact on the absorbance, and the spectra of the obtained samples have peaks characteristic of CDs. Considering CDs synthesized from the same material, the reached spectra have similar characteristics, but with CDs derived from different materials, the obtained samples have different peaks, which can be associated with the variation in the structure of the acquired CDs. As the temperature increases for samples synthesized from rapeseed pomace and potato peelings, the differences between the absorbance spectra decrease, but the difference between structures is significant. The size of CDs with polycrystalline structure (from apple pomace and from potato peelings) is similar to other CDs reported above (average 4–5 nm), while amorphic CDs are usually bigger. Regardless of the substrate used in this study, when excited with light in the near UV range, the obtained CDs exhibit fluorescence peaks that have similar characteristics to each other, and they are also within a similar spectral range (about 400–600 nm) as the studies cited above. The fwhm of the obtained samples is about 100 nm, which is similar to or lower than that observed in the cited studies, which is considered a favorable feature due to the narrower range of emitted radiation. It can be observed that fluorescence quantum yields of >50% are usually not obtained in thermal processes, but this is not always a necessary property for a given application (catalyst, drug carrier, fluorescence detection), so sometimes this value is not reported. The quantum yield of the obtained CDs characterized by the strongest fluorescence properties is 10–15%, which is slightly smaller than QY found in the literature for CDs synthesized by various methods from plant waste biomass. The efficiency of the synthesis process is usually not determined. In this paper, SY of 10–16.3% was obtained, which is close to the 10–19% present in the literature.

## 5 Conclusions

The possibility of synthesizing CDs from plant waste biomass with different sources of origin (apple, rapeseed, and potato) and associated different compositions by thermal degradation in a simple, solvent-free process was confirmed. The obtained CDs are characterized by similar fluorescence emission spectra and high quantum yields (max 14.5%). It is possible to achieve CDs with good fluorescence properties on a relatively high scale, from nonstandardized waste materials, as shown by the results presented here.

It was confirmed that the use of CDs synthesized by thermal degradation from plant waste allows for the optical detection of both simple substances, such as metal ions, and more complex compounds, such as ibuprofen. However, it was noted that surface-modified CDs exhibit better properties in terms of optical detection, including a lower limit of detection.

In the future, it would be desirable to study the process parameters and the effect of different solvents on the fluorescence properties of CDs derived by thermolysis of plant waste materials.

## Supplementary Material



## Data Availability

The authors confirm that the data supporting the findings of this study are available within the article and its Supporting Information. Raw research data are available upon request from the corresponding author, as they are part of doctoral research.

## Notes

Figures S1–S13 and Tables S1–S2, extended synthesis parameters, QS calibration curve, curves of dependence of fluorescence area vs absorbance of obtained CDs, full FT-IR spectra of waste biomass and products of its pyrolysis, deconvoluted X-ray diffractograms, and HR-TEM micrographs used for analyses (PDF)
