# Supplementary material for: Preparation of Biomass Waste-Derived Carbon Dots by the Thermal Degradation Process
Source: ACS Omega. 2025 May 27;10(22):22529–48. doi: 10.1021/acsomega.4c10119 (PMC12163701; doi:10.1021/acsomega.4c10119)
Supplement: Supplementary file 1 [file ao4c10119_si_001.pdf]

Supporting Information for
Preparation of biomass waste-derived carbon dots by the thermal degradation process

Wiktoria Matyjasik[a*], Krzysztof Matus[b], Olga Długosz[c], Jolanta Pulit-Prociak[c], Marcin Banach[c]

Corresponding author: Wiktoria Matyjasik (wiktoria.matyjasik@doktorant.pk.edu.pl)

[a] Cracow University of Technology, CUT Doctoral School, Faculty of Chemical Engineering and Technology, Department of Chemical Technology and Environmental Analytics, Warszawska St. 24, 31-155, Cracow, Poland

[b] Silesian University of Technology, Institute of Engineering Materials and Biomaterials, Konarskiego 18A, 44-100 Gliwice, Poland

[c] Cracow University of Technology, Faculty of Chemical Engineering and Technology, Department of Chemical Technology and Environmental Analytics, Warszawska St. 24, 31-155, Cracow, Poland

Supporting Information is presented on nine (9) pages (S1-S9), and contains 13 figures (Figure S1-S13 and 2 tables (Table S1-S2)

No.	T [°C]	t [min]	CDs labelling	d_{mavg} [%]	No.	T [°C]	t [min]	CDs labelling	d_{mavg} [%]
**1**	220	10	**Z-220**	8.5% ± 0.86%	8	220	30	**Z-220-30**	18.6%
**2**	240		**Z-240**	25.1% ± 1.47%	9	240		**Z-240-30**	39.6%
**3**	260		**Z-260**	40.9% ± 0.89%	10	260		**Z-260-30**	50.7%
**4**	280		**Z-280-10**	50.3% ± 2.75%	11	280		**Z-280-30**	53.9%
**5**	300		**Z-300-10**	53.1% ± 0.29%	12	300		**Z-300-30**	56.8%
**6**	320		**Z-320-10**	56.2%	13	320		**Z-320-30**	57.8%
**7**	340		**Z-340-10**	59.5%	14	340		**Z-340-30**	60.3%

Table S1. Summary of CDs extended synthesis parameters, labels of the obtained products and average weight loss of the material

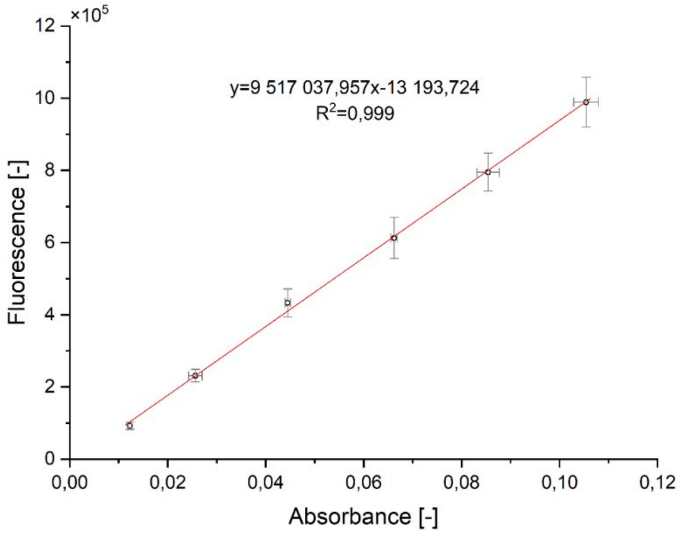

Figure S1. Calibration curve of the dependence of the fluorescence area on the absorbance of quinine sulfate (QS) in 0.5M H2SO4 at 350 nm.

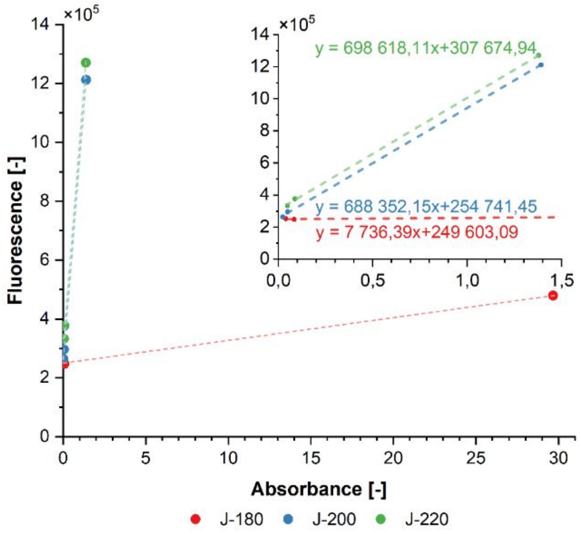

Figure S2. Curve of dependence of fluorescence area on absorbance of CDs obtained by thermal process from apple pomace

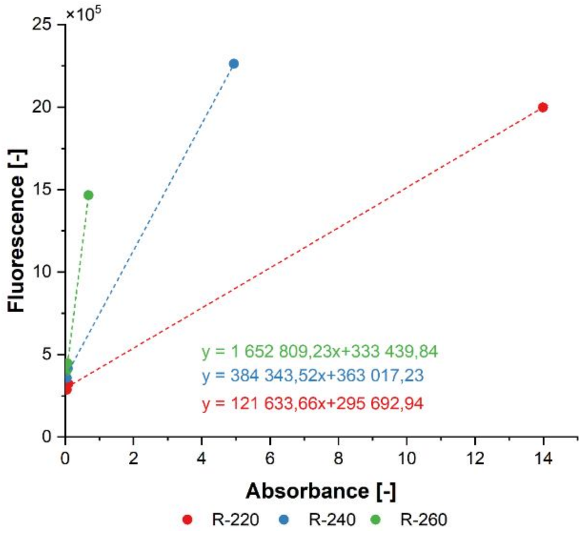

Figure S3. Dependence curve of fluorescence area on absorbance of CDs obtained by thermal process from rapeseed pomace

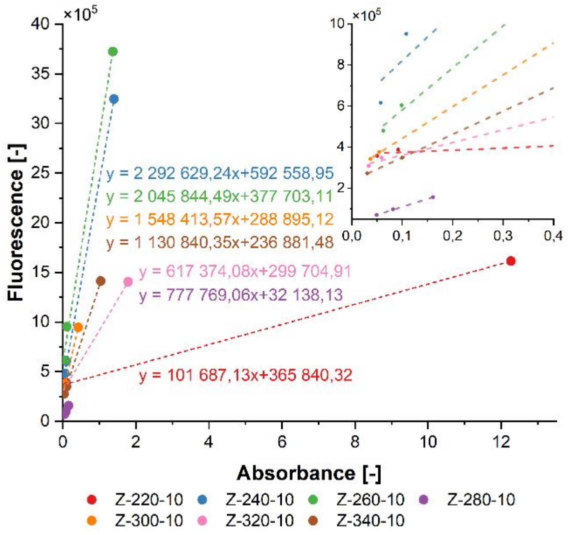

Figure S4. Curve of dependence of fluorescence area on absorbance of CDs obtained by thermal process from potato peelings (t=10 min)

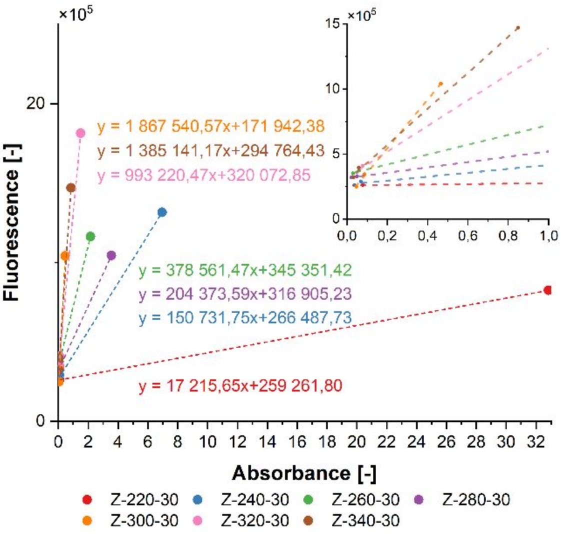

Figure S5. Curve of dependence of fluorescence area on absorbance of CDs obtained by thermal process from potato peelings (t=30 min)

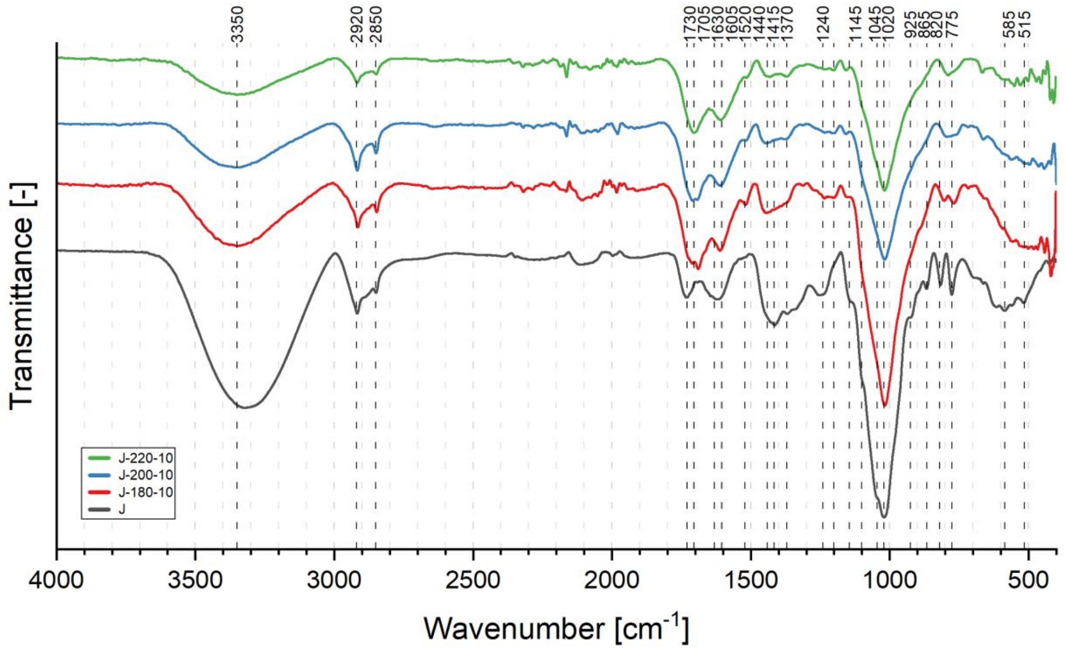

Figure S6. FT-IR spectra of apple pomace (J) and products of thermal degradation

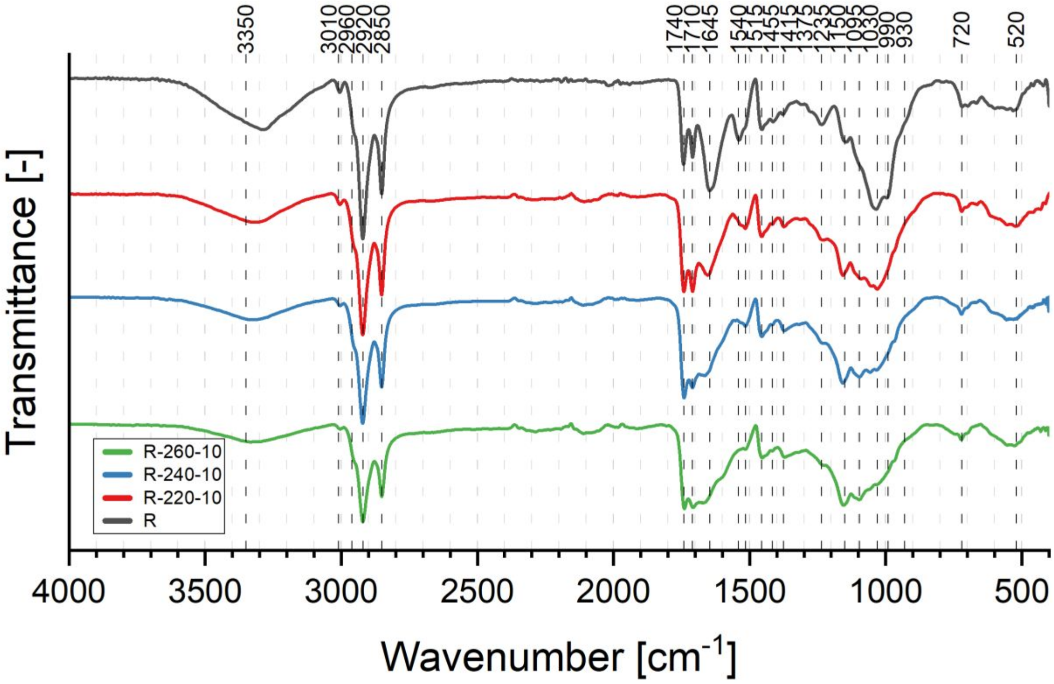

Figure S7. FT-IR spectra of rapeseed pomace (R) and products of its thermal degradation

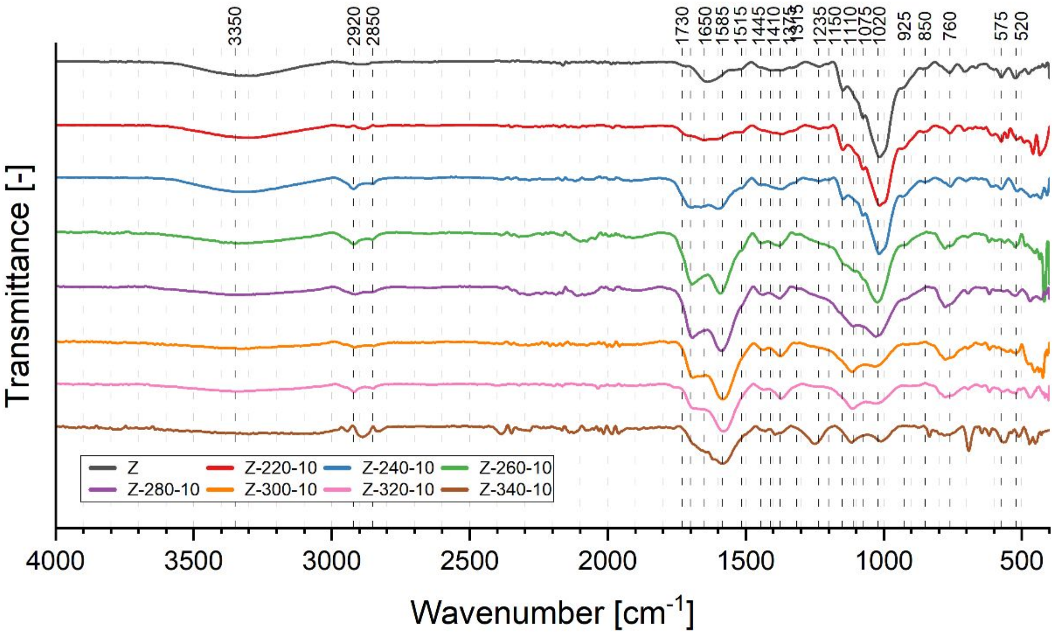

Figure S8. FT-IR spectra of potato peelings (Z) and products of its thermal degradation (t=10 min)

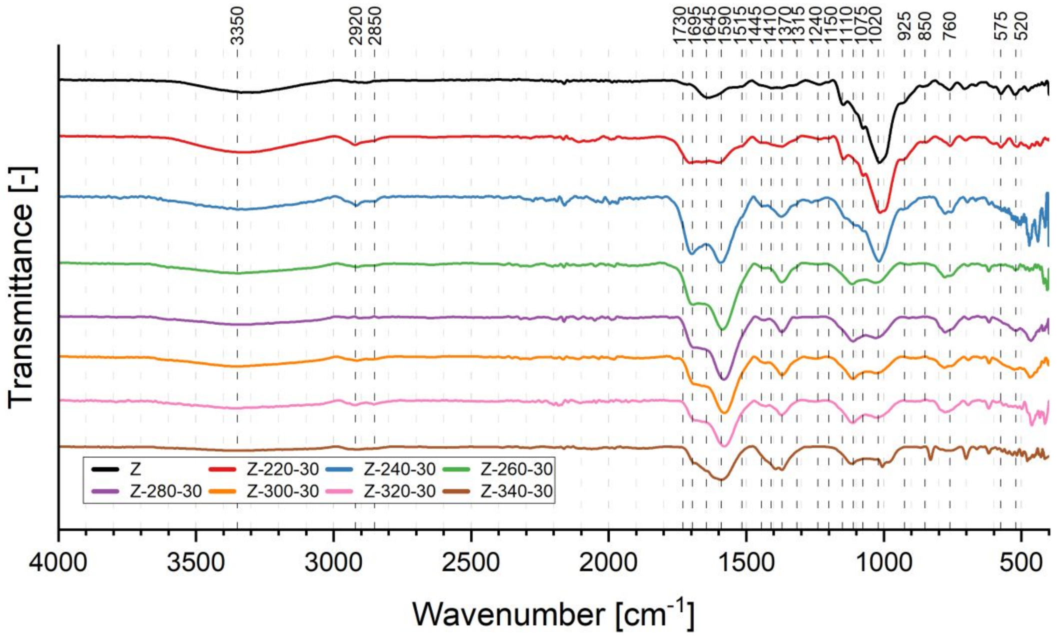

Figure S9. FT-IR spectra of potato peelings (Z) and products of its thermal degradation (t=30 min)

NaCl		
**J-220** 2Θ value [°]	**Z-260** 2Θ value [°]	**Plane**
27,35418	27,33892	111
31,69705	31,68183	200
45,44766	45,42674	220
53,86921	53,84856	311
56,48785	56,45629	222
66,24762	66,22104	400
75,31206	75,29289	420

Table S2. Values of 2Θ angle of NaCl residues in synthesized CDs

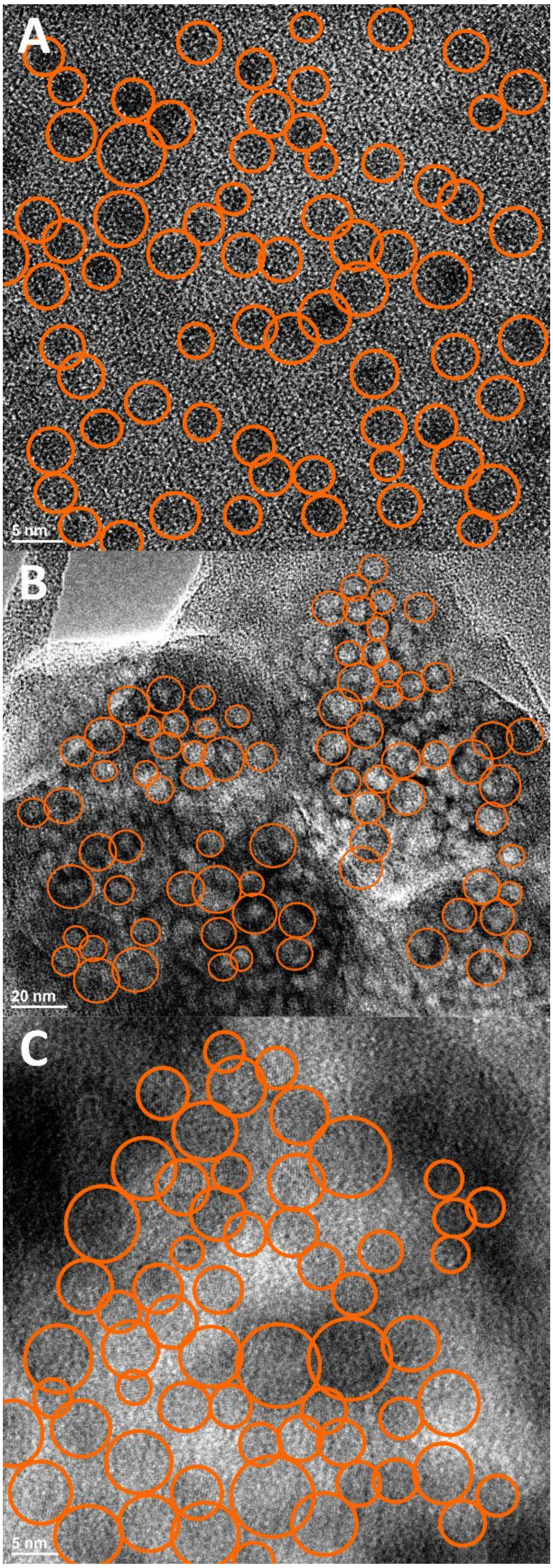

Figure S10. HR-TEM microphotographs of CDs obtained from the biowaste feedstock: apple pomace (A), rapeseed pomace (B) and potato peelings (C), with the counted particles circled

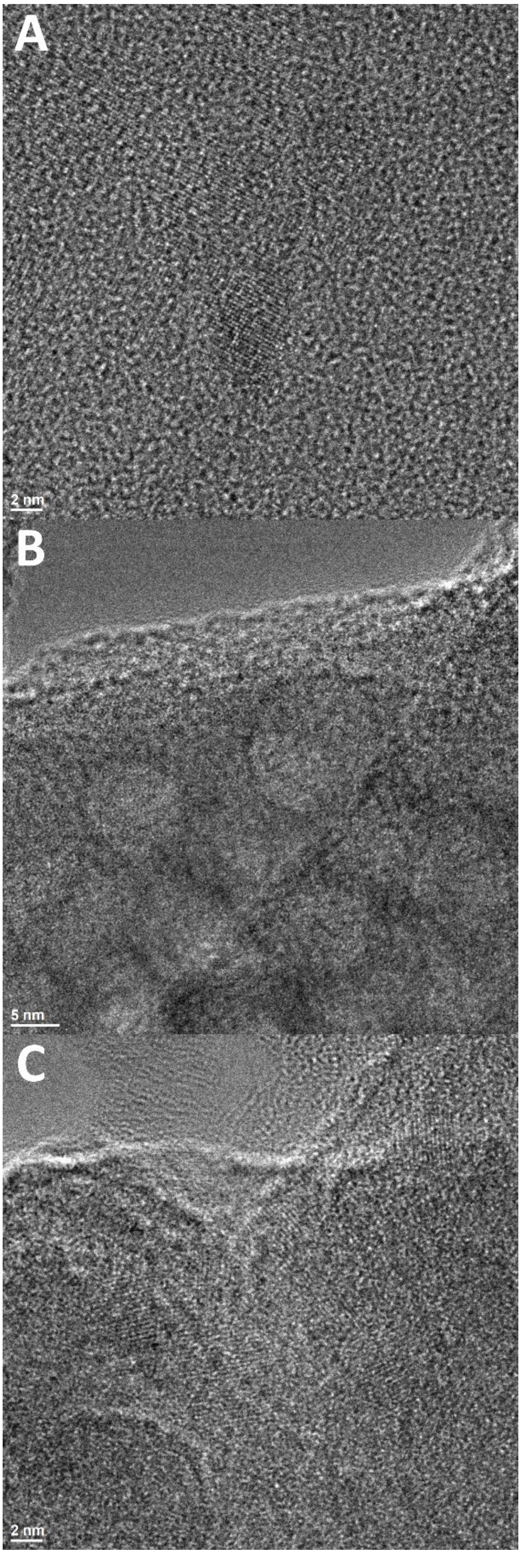

Figure S11. HR-TEM microphotographs of CDs obtained from the biowaste feedstock: apple pomace (A), rapeseed pomace (B) and potato peelings (C), for which crystal planes were determined

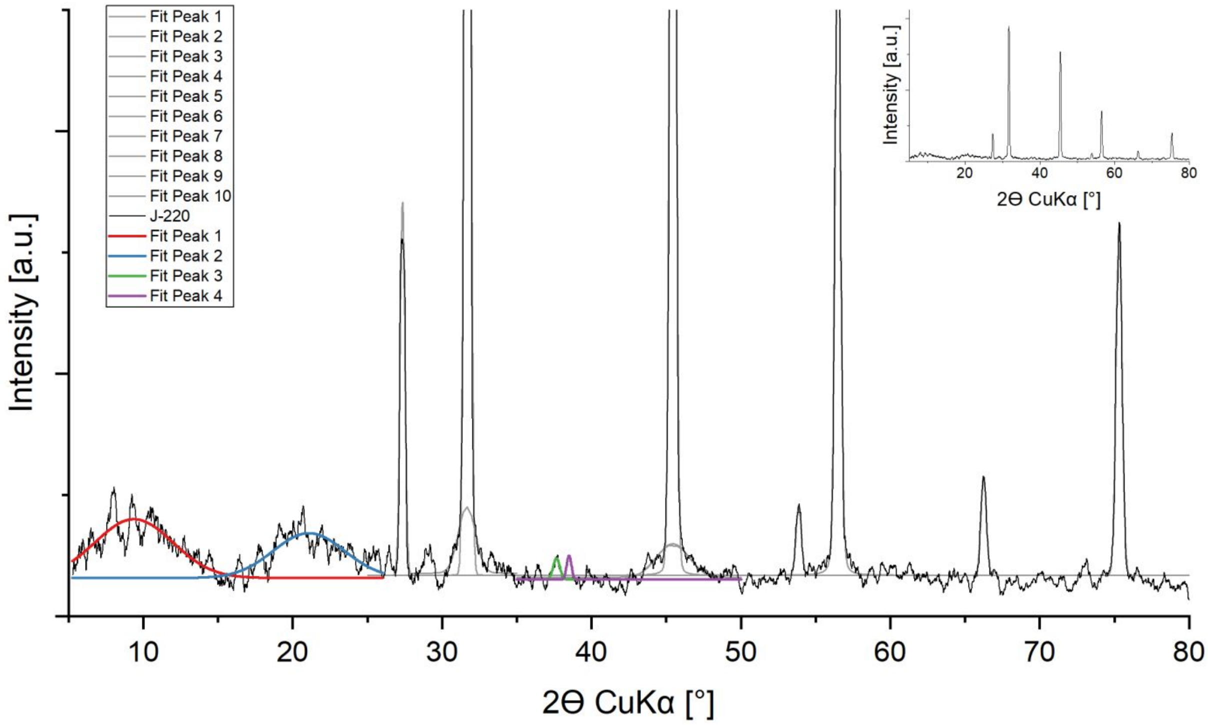

Figure S12. X-ray diffractogram after deconvolution of dried CDs sample J-220 derived from apple pomace

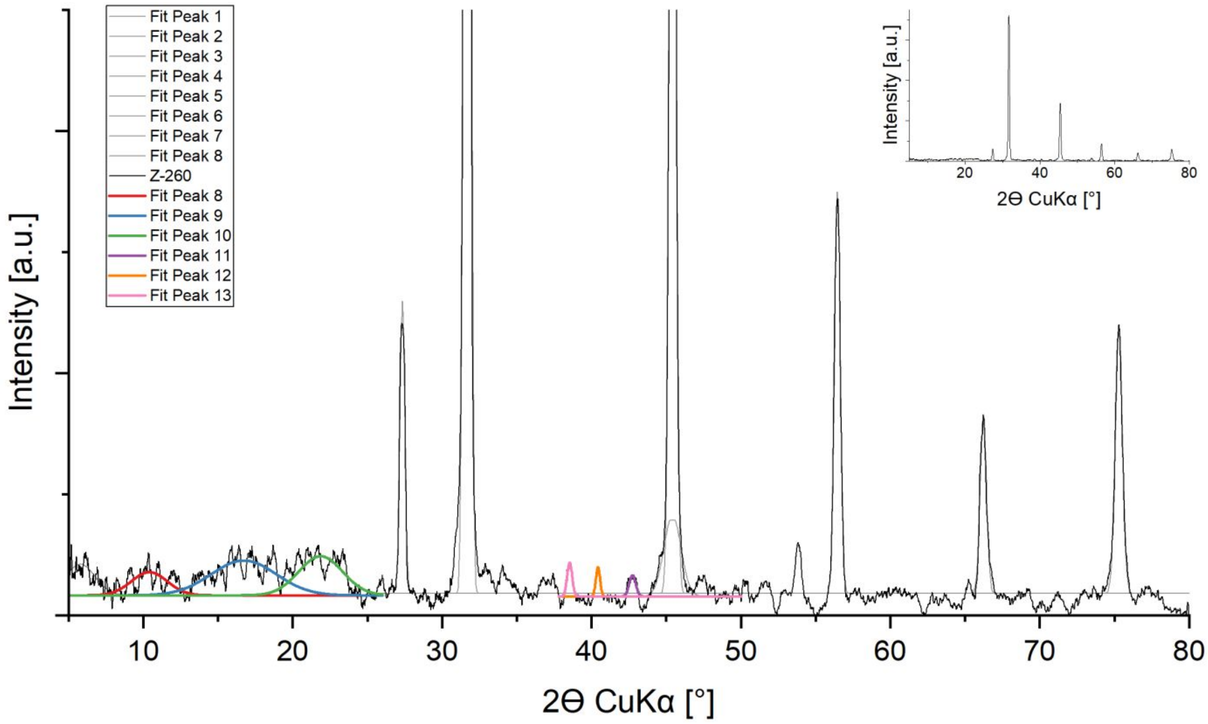

Figure S13. X-ray diffractogram after deconvolution of dried CDs sample Z-260 derived from potato peelings
